# Carroll’s Three-Stratum (3S) Cognitive Ability Theory at 30 Years: Impact, 3S-CHC Theory Clarification, Structural Replication, and Cognitive–Achievement Psychometric Network Analysis Extension

**DOI:** 10.3390/jintelligence11020032

**Published:** 2023-02-06

**Authors:** Kevin S. McGrew

**Affiliations:** Institute for Applied Psychometrics, 1313 Pondview Lane E, St. Joseph, MN 56374, USA; iqmcgrew@gmail.com

**Keywords:** intelligence, Carroll, Horn, Cattell, three-stratum theory, CHC theory, Gf-Gc, factor analysis, psychometric network analysis

## Abstract

Carroll’s treatise on the structure of human cognitive abilities is a milestone in psychometric intelligence research. Thirty years later, Carroll’s work continues to influence research on intelligence theories and the development and interpretation of intelligence tests. A historical review of the relations between the 3S and CHC theories necessitates the recommendation that the theories of Cattell, Horn, and Carroll be reframed as a family of obliquely correlated CHC theories—not a single CHC theory. Next, a previously unpublished Carroll exploratory factor analysis of 46 cognitive and achievement tests is presented. A complimentary bifactor analysis is presented that reinforces Carroll’s conclusion that his 3S model more accurately represents the structure of human intelligence than two prominent alternative models. Finally, a Carroll-recommended higher-stratum psychometric network analysis (PNA) of CHC cognitive, reading, and math variables is presented. The PNA results demonstrate how PNA can complement factor analysis and serve as a framework for identifying and empirically evaluating cognitive–achievement causal relations and mechanisms (e.g., developmental cascade and investment theories), with an eye toward improved cognitive–achievement intervention research. It is believed that Carroll, given his long-standing interest in school learning, would welcome the integration of theory-driven factor and PNA research.

## 1. Introduction

Carroll’s enduring legacy is captured by Beaujean’s (2015) statement, over 25 years later, that “the importance of his *Human Cognitive Abilities* book probably cannot be overstated. It is arguably one of the most influential works in the history of intelligence research” (p. 132).

This paper is dedicated to the memory of Jack Carroll. My 17-year informal mentor–mentee relationship with Carroll culminated in a week of collaborative activities where I lived and worked with Carroll in Fairbanks, Alaska, a little over a month before his passing on 1 July 2003. Much was accomplished during this week in May. Unfortunately, several of our planned collaborative tasks languished for approximately 20 years after his death. Several of these activities were completed for this paper, which is organized into three major sections.

First is a historical section that briefly describes the impact of Carroll’s 3S theory on CHC theory and intelligence test development and interpretation. This is followed by a clarification of the confusion surrounding the incorporation of Carroll’s 3S theory as part of CHC theory. This elucidation is necessary as semantic drift has beset the original intent of the term CHC theory. This section is, by necessity, a conflated mixture of personal and professional memories and reflections. The second section presents a previously unpublished Carroll exploratory factor analysis of a collection of 46 cognitive and achievement variables completed collaboratively with Carroll in May 2003. Consistent with Carroll’s (2003) last factor analysis publication, the final exploratory 46-test structure served as the starting point for a recently completed (summer 2022) bifactor confirmatory factor analysis (CFA) of the 46 tests. The last section extends the 46-test factor model via two psychometric network analyses (PNAs) of factor model-derived CHC composite variables. Ironically, the PNA of Carroll’s last known *g*-dominated factor analyses eschews the inclusion of a latent *g* factor. Yet, as explained herein, these non-*g* PNA models would likely be viewed favorably by Carroll as they are consistent with his long-held belief that some broad and narrow cognitive abilities are central to understanding the causal mechanisms of intelligence and school learning (Carroll 1993, 1998).

## 2. Carroll’s 3S Theory Impact on CHC Theory and Intelligence Testing

Carroll’s work is frequently melded with the theoretical work of Raymond Cattell and John Horn as the *Cattell–Horn–Carroll* (CHC) theory of cognitive abilities (McGrew 2009), the most widely recognized psychometric taxonomy of cognitive abilities (McGrew 2005, 2009; Schneider and McGrew 2012, 2018; McGill and Dombrowski 2019; Ortiz 2015; Wasserman 2019). Carroll’s 3S theory, as well as CHC theory, has had a major influence on psychometric theories of intelligence and intelligence test development, research, and interpretation practices.

The importance of Carroll’s 3S theory was recognized immediately by his intelligence research and theory peers (McGrew 2005). Horn characterized Carroll’s work as a “tour de force summary and integration“ comparable to Mendeleev’s periodic table of elements in chemistry (Horn 1998, p. 58). Jensen (2004) called it a magnum opus comparable to Hans von Bülow’s exclamation on his first reading of Wagner’s Die Meistersinger, “it’s impossible, but there it is!” (p. 4). Lubinski (2004) described the empirical foundation of Carroll’s work as “destined to be a classic” (p. 410). Later, in Carroll’s APA obituary, Lubinski (2004) described it as “among a handful of the best treatments ever published on individual differences in cognitive abilities. It is certainly the most definitive treatment of their nature and organization” (p. 44).

Two recent publications illustrate the enduring theoretical impact of Carroll’s (1993) work.^1^ Schneider and McGrew (2018) recently proposed incremental revisions to CHC theory from nascent signals present in Carroll’s book. These included (1) the division of Glr into Gl and Gr domains, (2) the addition of a broad general emotional intelligence (Gei) domain, (3) the bifurcation of perceptual speed (Gs-P) into two perceptual speed sub-factors (P-Search/Scanning and P-Comparison/Pattern Recognition), and (4) the need to subcategorize Gv as per large-scale navigation and small-scale spatial abilities.^2^ More recently, Whilhelm and Kyllonen (2021) articulated how the strengths and limitations of Carroll’s work can serve as a springboard for new research focusing on such topics as (1) the central importance of working memory for Gf and recognizing Gf content facets, (2) reconceptualizing Gc as more akin to the Cattell-like notion of a general capacity (*g_c_*) that includes, aside from verbal abilities, other forms of acquired knowledge, achievement, and domain-specific expertise, (3) better understanding the complex nature, measurement, and scaling of speed factors (Gs, Gt, and Gps), (4) the relation between idea production (Gr) and the emerging societal need to better understand and foster creativity, and (5) the need to incorporate social, emotional, and collaborative intelligence constructs in a more comprehensive intelligence framework. Whilhelm and Kyllonen (2021) proposed five research strategies for moving the study of intelligence forward, including the integration of cognitive and non-cognitive traits (e.g., motivation, interests, and personality) consistent with contemporary notions of Snow’s aptitude trait complexes (Ackerman 1996, 2018; Corno et al. 2002; McGrew 2022).

Carroll’s 3S theory stands on its own merits in the space occupied by intelligence scholars. However, it could be argued that his 3S theory’s impact on the practice of intelligence test development and interpretation is equal to, or greater than, his impact on theories of intelligence. Why?

Using Rappaport et al.’s (1945–1946) *Diagnostic Psychological Testing* as an approximate start date, the clinical interpretation of intelligence tests has been characterized as progressing through four overlapping “waves” of interpretation practices (Kamphaus et al. 2012) spanning 40 to 50 years. These overlapping waves produced both missteps and incremental progress, reflecting a combination of ad hoc psychometric considerations and the retrofitting of older theories of cognitive abilities to intelligence test data (Schneider and Flanagan 2015). *Kaufman’s Intelligent Testing with the WISC-R* (Kaufman 1979) was prescient in articulating the fourth wave (circa 1980′s to present)—the application of emerging theories of intelligence to test interpretation. Luria’s neuropsychological theory influenced both the interpretation of existing tests and the development of new Luria-based cognitive tests (*Kaufman Assessment Battery for Children*, Kaufman and Kaufman (1983); *Cognitive Assessment System*, Naglieri and Das (1997)). Early psychometric-based theories (e.g., Spearman’s *g*, Cattell’s *g_f_-g_c_*; and Thurstone’s primary mental abilities) sowed the seeds for the primarily factor-analytic-based Cattell–Horn Gf-Gc and Carroll 3S theories. These seeds quickly bloomed when the practical import of Carroll’s work, coupled with Carroll’s (1993) recognition of Cattell and Horn’s Gf-Gc theory, was synthesized under the umbrella of CHC theory. The appearance of Carroll’s work signaled the end of the lengthy quest for a defensible and empirically based theoretical framework to guide intelligence test development and interpretation.

The emergence of CHC theory produced multiple positive outcomes. The existing intelligence theory–research–practice testing gap quickly narrowed (Flanagan et al. 2000; McGrew and Flanagan 1998; McGrew 2005; Ortiz 2015). CHC theory became the cornerstone for the development and interpretation of most major individually administered intelligence tests (Benson et al. 2018; Caemmerer et al. 2020; Daniel 1997; Flanagan et al. 2013; Kaufman 2009; Keith and Reynolds 2010; McGrew and Flanagan 1998; Wasserman 2019). CHC theory also influenced research in other fields of psychology (e.g., neuropsychology; brain network research; behavioral genetics; e.g., see Barbey 2018; Jewsbury et al. 2017; Procopio et al. 2022) and other non-psychology fields (e.g., the military; *Atkins* intellectual disability death penalty cases; and computer science education and the internet; Schneider and McGrew 2018).

### 2.1. Carroll’s 3S Theory’s Connection with CHC Theory: An Arranged Marriage of Convenience

The rapid infusion of Carroll’s work in the intelligence testing literature was, in large part, initiated and accelerated by regular interactions between the theorists Carroll and Horn and the Woodcock–Johnson—Revised (WJ-R; Woodcock and Johnson 1989) and Woodcock–Johnson III (WJ III; Woodcock et al. 2001) test author teams (Benson et al. 2018; McGrew 2005; McGill and Dombrowski 2019; Wasserman 2019). These interactions stemmed from the documented serendipitous March 1986 Dallas, Texas, “meeting of the minds” (John Carroll, John Horn, and Richard Woodcock) that gave eventual birth to the first individually administered cognitive test battery (WJ-R) organized as per the broad strokes of the Cattell–Horn Extended Gf-Gc theory of intelligence (McGrew 2005; Kaufman 2009; Ortiz 2015). Seven years later, Carroll (1993) concluded that the Cattell–Horn Gf-Gc theory “appears to offer the most well-founded and reasonable approach to an acceptable theory of the structure of cognitive abilities” (p. 62). Carroll’s endorsement of the Cattell–Horn Gf-Gc theory was viewed as a signal in the field of intelligence testing that his 3S model was, in terms of broad ability dimensions, like Cattell and Horn’s Gf-Gc framework. Carroll’s work provided an authoritative independent stamp of approval, cementing the birth of what is now known as CHC theory.

In 1999, Woodcock brokered the CHC umbrella term with Horn and Carroll for practical reasons (McGrew 2005)—to facilitate internal and external communication regarding the theoretical model of cognitive abilities underlying the then-overlapping test development activities (and some overlapping consultants, test authors, and test publisher project directors; John Horn, Jack Carroll, Richard Woodcock, Gale Roid, Kevin McGrew, Fred Schrank, and John Wasserman) of the Woodcock–Johnson III and the Stanford Binet–Fifth Edition by Riverside Publishing (Kaufman 2009; McGrew 2005; Wasserman 2019). There was no formal proclamation in a psychology journal where Cattell, Horn, and Carroll blessed the pragmatic union of their respective theories. In some respects, this union was more akin to an arranged marriage.

Several visible activities of the WJ III authors contributed to the establishment of CHC theory in the intelligence literature (McGill and Dombrowski 2019; Wasserman 2019). Woodcock’s (1990) joint CFA of the WJ-R with five other intelligence batteries, as per the Cattell–Horn Gf-Gc theory, raised awareness of the Cattell–Horn broad abilities in the intelligence testing community. Woodcock suggested the concept of “crossing batteries” to measure all major broad cognitive abilities. Subsequently, McGrew (1997) proffered an integrated Cattell–Horn–Carroll framework for test interpretation. According to McGrew (2005), the first formally published definition of CHC theory was presented in the WJ III technical manual (McGrew and Woodcock 2001), and CHC was featured prominently in the WJ III test manuals (McGill and Dombrowski 2019). McGrew was also involved in the formative stages of the development of the CHC cross-battery assessment method (Flanagan and McGrew 1997; McGrew and Flanagan 1998; Flanagan et al. 2000) popularized by Flanagan and colleagues (Flanagan et al. 2013). Finally, it may have been McGrew’s (2009) editorial in *Intelligence* (“CHC theory and the human cognitive abilities project: Standing on the shoulders of the giants of psychometric intelligence research”) that was in large part responsible for the accelerated infusion of CHC theory into the intelligence literature. In McGill and Dombrowski’s (2019) description of the evolution of CHC theory, they referenced a bibliometrics analysis of the journal *Intelligence* that found McGrew’s (2009) editorial to be the most cited article in the journal over an eight-year period (2008–2015). The CHC’s foothold in the intelligence literature was further consolidated by a series of CHC theory book chapters (McGrew 2005; Schneider and McGrew 2012, 2018).

### 2.2. Criticisms of Carroll’s 3S and CHC Theory

As with any paradigm-shifting event in science, Carroll’s 3S theory (and affiliated CHC theory) has not escaped criticism. Consistent with Carroll’s reputation as a scholar with unquestionable scientific integrity,^3^ Carroll, after acknowledging his gratification with the largely effusive initial reviews of his work by his peers, expressed disappointment that the largely positive reviews “didn’t tell me what I wanted to know: What was wrong with my book and its ideas, at least what might be controversial about it, for surely it dealt with a field that has abounded in controversy” (Carroll 1998, p. 6). Carroll, being Carroll, proceeded to publish a self-critique of his work (Carroll 1998).

The formal critiques Carroll so desired did not materialize until approximately 20 years after his book, approximately 10 years after his passing. It is beyond the scope of this paper to review the details of the published critiques of Carroll’s work or CHC theory (see Benson et al. 2018; McGill and Dombrowski 2019; Wasserman 2019; Whilhelm and Kyllonen 2021). One salient criticism of CHC theory (and, by association, Carroll’s 3S theory) is relevant to the current historical narrative—the “circular” or “enmeshed” relationship between CHC theory and the WJ-R, WJ III, and WJ IV test authors (McGill and Dombrowski 2019; Wasserman 2019). This criticism states that the Cattell–Horn Gf-Gc or CHC theories were used to design the tests and organizational structures of the WJ-R, WJ III, and WJ IV, and the norm data from these test batteries were then used to verify the validity of the theoretical models. I recognize and acknowledge the gist of these criticisms during the formative years of CHC intelligence research. However, this criticism is now moot. CHC theory is, today, the joint property of the scientific community of intelligence scholars and those in the applied field of intelligence testing—a unique informal research–practice union. Furthermore, McGill and Dombrowksi (2019) acknowledged that “to be fair, there have been a number of CFA studies in which a CHC structure has been found to fit various datasets well (see Schneider and McGrew 2012)” (p. 223; also see McGrew (2005) and Schneider and McGrew (2018)). More recently, Caemmerer et al. (2020), using a sophisticated missing data confirmatory factor analysis method of subtests from the norming or linking of samples from six different intelligence test batteries (n = 3900+), found support for the validity of the CHC theory and the CHC classifications for all but 1 of the 66 tests.

### 2.3. A Recommended Name Change: CHC Theories—Not CHC Theory

The origin and evolution of the term CHC theory have been murky (McGill and Dombrowski 2019). A salient problem is the name itself—CHC theory. As explained above, CHC theory originated from the pragmatic need to reference the overarching similarities of the Cattell–Horn Gf-Gc and Carroll 3S theories. It was never intended to convey that Cattell, Horn, and Carroll had reached a formal rapprochement regarding all matters concerning the structure of human intelligence. Horn and Carroll had a long-standing difference of opinion regarding the nature and essence of *psychometric g*, which is defined, in this paper, as the statistical extraction of a latent factor (via factor analysis) that accounts for the largest single source of common variance in a collection of cognitive abilities tests. In contrast, *theoretical g* refers to the yet-identified underlying biological brain-based mechanism(s) that produces psychometric *g*. In McGrew’s (2009) *Intelligence* CHC theory article, it was stated “there are remarkable similarities between the Carroll 3S and Cattell–Horn Gf–Gc theories, so much so that a single umbrella term (viz., the *Cattell–Horn–Carroll (CHC)* theory of intelligence) was proposed to reflect the *broad stroke communality of these two most prominent theoretical models*” (p. 2; emphasis added). Additionally, four fundamental differences between the Cattell–Horn and Carroll models were elucidated. It appears that McGrew (2009) failed to make it sufficiently explicit that the original intent of the CHC term was to function as an informal broad umbrella term—*not to indicate a strong consensus among the three theorists under the common label*. The use of the term CHC theory was embraced with considerable enthusiasm in the intelligence theory and testing literature (Wasserman 2019), so much so that the “big tent” ecumenical meaning of the original term was lost. The ability of anyone associated with the origin of CHC theory to control the narrative of the CHC label quickly vanished. The CHC theory train had left the station. In the process, the primary differences between Carroll’s 3S conceptualization of the structure of cognitive abilities, when contrasted with Cattell and Horn’s unique contributions, were obscured. It is time to clarify the record.

Prior attempts to correct this misunderstanding were made in two CHC theory update chapters (Schneider and McGrew 2012, 2018). Schneider and McGrew (2018) stated that “the Cattell–Horn–Carroll theory of cognitive abilities (CHC theory) is a comprehensive taxonomy of abilities embedded in *multiple over-lapping theories* … *It has a “big-tent” mindset, tolerating ambiguities and disagreements wherever there are reasonable grounds for disagreement (e.g., the nature of general intelligence)* … Our hope for CHC theory is that it provides *a common framework and nomenclature* for intelligence researchers to communicate their findings *without getting bogged down in endless debates about whose version of this or that construct is better*” (p. 73; emphasis added).

A primary reason for this clarification is to fulfill my personal obligation to Carroll (from the May 2003 week-long discussions when I lived and worked with Carroll during his retirement while he lived in the home of his daughter and her family in Fairbanks, Alaska). Carroll acknowledged his involvement in the origin of the informal CHC umbrella term agreement (Carroll 2003), but was vexed that the term CHC theory had been so rapidly infused into the literature and, more importantly, incorrectly implied that he, Cattell, and Horn had agreed to a formal union of theories. I agreed to help clarify his position in a planned coauthored revision of Carroll’s 3S model chapter originally published in Flanagan et al. (1997). His passing just over a month later ended this plan. Furthermore, when researching the history of the origin of the CHC label for this article, after communicating with multiple individuals who were present at the time, I learned, for the first time, that Horn had also expressed a degree of exasperation with the message conveyed by the singular CHC term as soon as a few months after Woodcock brokered the term. Having worked with (and being informally mentored by) both Carroll and Horn during the WJ-R and WJ III revisions and related cognitive ability projects for nearly 17 to 20 years, respectively, there is a clear need to honor their memories by clarifying the meaning of CHC theory…to CHC theories.

The most salient difference between the three related CHC theories is the presence (or absence) of a higher-order general intelligence factor or ability (*g*). Horn was a staunch anti-*g* proponent who believed that psychometric *g* was nothing more than an emergent property statistical abstraction (McGrew et al. 2023)—it did not represent a true ability or mechanism in the human brain (Horn 1998; Horn and Noll 1997; McArdle 2007; McArdle and Hofer 2014; Ortiz 2015). Conversely, Carroll was a staunch proponent of psychometric *g* as possibly representing some form of biological substrate present in individuals that influences the speed and efficiency of information processing (Carroll 1991, 1993, 1996, 2003). However, often ignored in publications citing Carroll’s strong psychometric *g* position is Carroll’s admission that psychometric *g* might not represent a real theoretical or psychological construct. Examples include the following Carroll statements in three publications after his seminal work “the question remains whether a unitary *g* exists, or whether a factorially produced *g* is merely a *mathematical artifact*” (Carroll 1998, p. 13; emphasis added); “What has *not* been adequately demonstrated and proven at this time is that *g* is a ‘true ability” independent of more specific cognitive abilities defined by various types of psychological tests and observations” (Carroll 1997, p. 151, emphasis added); the “continued psychological and even philosophical examination of the nature of factor *g* is a *must*” (Carroll 2003, p. 19). These statements suggest that Carroll was aware, but did not make it sufficiently clear, that much of the intelligence structural analyses research literature suffers from the problem of conflating psychometric and theoretical *g* (Fried 2020; McGrew et al. 2023).

Occupying a more middle ground is Cattell, who eschewed his mentor’s (Spearman) singular *g* in favor of multiple *general capacities*. As per Wasserman’s (2019) summary of Cattell’s Triadic theory, “Cattell believed that there are four *general capacities* (fluid reasoning, memory, retrieval fluency, and speed); a handful of provincial powers corresponding to sensory and motor domains (e.g., visual, auditory, olfactory, gustatory, tactile, and cerebellar); and a large number of environmentally developed capacities he called agencies (e.g., mechanical ability, literacy, numeracy, and so forth). One developed agency, crystallized intelligence, is so broad in scope that it behaves like a general capacity (Cattell 1987)” (p. 253). Cattell’s general capacities, which replace Carroll’s dominant psychometric *g* factor, are those abilities (fluid intelligence—*g_f_*; crystallized intelligence—*g_c_*, memory—*g_m_*, retrieval fluency—*g_r_*, and speed—*g_s_*)^4^ involved across most all cognitive tasks and reflect limiting properties, parameters, or constraints of the action of the whole brain (Cattell 1987).

Also missing from the CHC theory literature is an accurate attribution of the origins of the three-stratum hierarchical model of cognitive abilities. Carroll did not originate the term stratum and attributed the distinction between the *order* and *stratum* of factors to Cattell (1971). Vernon’s (1950) hierarchical group factor theory, although not using the term stratum, differentiated cognitive ability factors as per a three-level hierarchy with *g* at the apex, two major group factors (verbal–numerical–educational, *v:ed*; and practical–mechanical–spatial–physical, *k:m*), and, subsequently, a variety of minor group factors (e.g., number, verbal, spatial, mechanical information, and manual). It may have been Alexander (1934) who, although factoring a more limited array of ability measures, first articulated a three-level hierarchical model (general intelligence (*g*); practical (*p*) and verbal (*v*); mechanical (*m*), number (*n*), and reasoning (R)) (Vernon 1950). Alexander’s (1934) work was prescient of the distinguishing of factors as per *breadth* (i.e., stratum), by which “we mean the number of life situations in which a factor plays some part (whether more or less important)” (p. 82).

There is a long-overdue need to correct the misunderstanding surrounding the meaning of the CHC theory label. Analogous to how the term “information processing” theories or models serves as a broad umbrella for similar or competing theories, CHC represents several related (yet different) theoretical structural models of cognitive abilities (McGrew 2005). It is recommended that CHC theory henceforth be referred to as CHC theories. In addition, Cattell’s notion of multiple general capacities should be revisited and empirically compared with the Carroll (there is *g*) and Horn (there is no *g*) structures. CHC theories clearly build on the shoulders of multiple psychometric intelligence giants (McGrew 2009).

### 2.4. Summary Comments

In summary, the origin and evolution of CHC theory (and Carroll’s 3S theory’s role in the theory) as described herein and elsewhere has been a non-linear, messy process. This is often the nature of cumulative science, which is replete with unanswered questions, critiques, anomalies, serendipitous events, steps forward and backward, misunderstandings, and unintended consequences based on noble scientific intentions. I concur with the apt description of Wasserman (2019) that “contrary to idealistic expectations about how science advances, there may be no defining moment when CHC is considered fully and consensually confirmed or disconfirmed. CHC itself will be amended, fixed and patched whenever possible… For better or worse, this seems to be how science works” (p. 262). Although Carroll and Horn had become vexed by the use of the singular CHC label, I agree with Ortiz (2015) that “Cattell, Horn, and Carroll would all likely take heart in knowing that their burning interest in furthering an understanding of intelligence and human cognitive abilities has been passed on intact to the current generation of psychological scientists. CHC theory has grown up, and it would be safe to assume that Cattell, Horn, and Carroll would likely be very proud to know that their goals, their ideas, and their passion are still alive and kicking in CHC theory to the present day” (p. 226). Notwithstanding, it is henceforth recommended that the three related theories of Cattell, Horn, and Carroll be reframed as a family of obliquely correlated CHC theories, reflecting their strong similarities, as well as fundamental differences.

## 3. A Replication and Extension of Carroll’s (2003) Comparison of the Three Primary Structural Models of Cognitive Abilities

### 3.1. Carroll’s (2003) Analyses That Supported His Standard Multifactorial 3S View of Intelligence

Carroll’s (2003) last published factor analyses were of the 16- and 29-test kindergarten-through-adult age-range test correlation matrices reported in the WJ-R technical manual (McGrew et al. 1991). He completed these analyses with the latest version of the EFA-Schmid/Leiman software (EFA-SL) used in the analyses presented in his seminal work. Carroll used the EFA-SL solutions as inputs for the specification and evaluation of bifactor CFA (LISREL) models to estimate and evaluate the final orthogonal factor loadings. The purpose of his 2003 analyses was to compare and evaluate three different views of the structure of cognitive abilities. Carroll described the three models as the (1) *standard multifactorial view*, which is Carroll’s hierarchical *g* 3S model; (2) *limited structural analysis view*, typically associated with Gustafsson and colleagues that includes a higher-order *g*-factor that is either highly correlated or isomorphic with the broad Gf factor; and (3) *second-stratum multiplicity view*, the model most often associated with Horn that does not include a higher-order *g-*factor and instead posits correlated broad factors. These three views are hereafter referred to as the *Carroll 3S*, *Gustafsson Gf = g*, and *Horn no-g* models, respectively.

In his WJ-R analyses, aside from a large *g*-factor, Carroll identified eight (16-test analyses) and nine (29-test analyses) factors he labeled as per the broad CHC ability terminology used (at the time) by the WJ-R author team. His analyses supported a higher-order *g*-factor and eight broad factors (viz., Gl, Gwm, Gs, Ga, Gv, Gc, Gf, and Gq).^5^ A tentative Language (Lang) factor in the 29-test analyses was identified and was characterized as needing further research. Carroll (2003) concluded that his two analyses “confirm the classical, standard multifactorial model of the higher-stratum structure [Carroll 3S] … [and] tend to discredit the *limited structural analysis view* [Gustafsson Gf = *g*] and the *second-stratum multiplicity view* [Horn no-*g*]” (p. 17). A close reading finds that Carroll did not completely reject the Gustafsson *Gf = g* model as “the low *Gf* factor loadings most likely indicate that the factor *Gf* is inherently difficult to measure reliably independently of its dependence on *g* (as indicated by the high *g* loadings for these tests). This may account for the finding by Gustafsson … that it is often difficult to distinguish *Gf* from *g*” (Carroll 2003, p. 14). Carroll (2003) was dismissive of the Horn no-*g* model when he concluded that “it would be difficult to argue that the *g* factors yielded by the two analyses are different, even though they involve different second-order factors … I cannot regard Horn’s comment as a sound basis for denying the existence of a factor *g*, yet he succeeded in persuading himself and many others to do exactly this for an extended period of years” (p. 19).

### 3.2. Confirmation of Carroll’s 3S Multifactorial View with 46 WJ III Tests

One central task during the May 2003 work week with Carroll was the collaborative completion of a Carroll EFA-SL factor analysis of the ages 14–19 correlation matrix of 46 WJ III tests (*n* = 1618) reported in the WJ III technical manual (McGrew and Woodcock 2001). The ages 14–19 sample was a subsample of the complete WJ-III norm sample, which was a nationally representative sample of 8818 participants from age 2 through 90 plus. The WJ-III Technical Manual reports that the norm sample was matched to the 2000 U.S. Census for the demographic variables of geographic region, community size, sex, race, educational level, and occupation. Detailed demographic characteristics are provided in the WJ-III Technical Manual. Descriptions of the WJ III tests are presented in Supplementary Material Section S2.

The procedures described in Carroll’s 1993 methodology chapter (Chapter 3) guided the analysis. The best available existing copy of the final model output (original date 05-29-03), as well as a more readable, reorganized version of the output, are presented in Supplementary Materials Section S3. The final solution identified 10 interpretable factors—9 first-order factors and 1 large general intelligence (*g*) factor. Based only on the findings of the EFA-SL analysis of the 46 WJ III tests, Carroll believed that the analyses supported his conclusion (Carroll 2003) that the Carroll 3S model, when compared with the Gustafsson *Gf = g* and Horn no-*g* models, was more valid.

With the unfortunate passing of Carroll on 1 July 2003, a little over a month after the McGrew–Carroll EFA-SL analysis was completed, the second planned step (bifactor CFA using the EFA-SL model as a starting point) was never completed. This delinquent bifactor CFA was recently completed (summer of 2022) for the current paper. The analyses and results are reported next.

The salient WJ III test loadings on the 10 EFA-SL factors served as the starting point for the specification of a bifactor CFA model. Details regarding the decisions made during the CFA analyses are included in Supplementary Materials Section S4. The statistical analysis was completed with the open-source *JASP* (v 0.16.3; JASP Team 2022) factor analysis software program module supported by the University of Amsterdam (https://jasp-stats.org/ accessed on 4 October 2021). Although the results presented can be interpreted to reflect on the structural validity of the WJ III test battery, in this paper, the interpretations are restricted to the theoretical questions investigated by Carroll (1993, 2003). Furthermore, comparisons of the current McGrew–Carroll 2003/2022 WJ III 46-test results (hereafter referred to as the *MC analysis*) are only made with Carroll’s 29-test WJ-R analysis results (hereafter referred to as the *Carroll analysis*), not with the 16-test analysis. Although my current and evolving views regarding psychometric and theoretical *g* are not 100% consistent with Carroll’s (see McGrew et al. 2023), to the best of my ability, I present what I believe would be interpretations consistent with Carroll’s thinking of the MC analysis results. The final bifactor CFA model results are presented in Table 1.

Although the Carroll and MC analyses differ in the number of test indicators of broad abilities, in general, the 11 MC analysis factors (1 *g* and 10 broad) presented in Table 1 are consistent with the Carroll analyses factors (1 *g* and 9 broad; see Carroll’s 2003 chapter Table 1.5) and thus, as explained below, support his 2003 conclusions regarding the validity of his 3S model.

Both the MC and Carroll analyses produced prominent *g* factors. The MC analysis *g* factor (O2-*g* in Table 1) is a large general factor with factor loadings ranging from .25 to .86, while the Carroll analysis *g* factor had loadings ranging from .28 to .78. Approximately 76% (*n* = 35 of 46) of the MC analysis *g* factor loadings were greater than or equal to .50, while 62% (*n* = 18 of 29) of the Carroll analysis *g* factor loadings met the same criterion. The MC analysis *g* factor had a larger percentage of tests with high *g* loadings. The MC analyses provide strong support for a dominant *g* factor as per Carroll’s 2003 3S conceptualization of intelligence. Interestingly, in both analyses, the strongest loading tests on the large *g* factor were primarily (or were mixed) tests of acquired knowledge (Gc, Gq, and Grw; like Cattell’s *g_c_*), Gf (especially in the MC analysis, where four Gf tests had *g* loadings ≥ .70+), and, to a lesser extent (in the MC analysis), tests of Gwm or Gl.

Eight of the MC analysis factors appear to be broader (defined by a larger set of tests representing a more diverse range of narrow abilities) analogs of the same broad factors in the Carroll analyses (viz., Gc/Gc, Gl/Glr, Gsc/Gs, Gf/Gf, Gsm/Gwm, Gq/Gq, Gv/Gv, and Ga/Ga). For example, as reflected in Table 1, the MC analysis Gc factor was defined by the Picture Vocabulary (.65), Verbal Comprehension (.45), Oral Comprehension (.14; named Listening Comprehension (LISCM)^6^ in the WJ-R Carroll analysis = .*42*), and Academic Knowledge tests (.26, which is the combination of the similar Science (SCIENC = *.49*), Social Studies (SOCSTU = *.49*), and Humanities (HUMANI = *.45*) tests in the Carroll analysis). The MC Gc factor also included Gc-consistent salient loadings (for tests absent from the Carroll analysis) that were clear indicators of Gc (General Information = .34; Reading Vocabulary = .20), and with lower, yet still Gc-consistent, loadings for Story Recall (.14), Memory for Sentences (.14) and Passage Comprehension (.08). Additionally, Picture Vocabulary (PICVOC = *.53*), Oral Vocabulary (ORALVO = *.37*), and Verbal Analogies (VBLANL = *.16*) all had salient loadings on the Carroll analysis Gc factor. The composition and test factor loadings for the respective Gsm/Gwm and Ga/Ga factors in the two analyses clearly represent similar broad cognitive dimensions across both analyses and, thus, do not warrant detailed discussion.

MC analysis factors that, upon first inspection, suggest possible broad factors that differ from the Carroll analyses (Gl/Glr; Gv/Gv) are interpreted as similar when the specific tests loading on the factors are reviewed. For example, in both the MC and Carroll analyses, the respective Gl and Glr factors both had relatively strong loadings for the Memory for Names (MC analysis = .48; Carroll analysis MEMNAM = *.70*) and Visual–Auditory Learning (MC analysis = .44; VISAUD = *.34*) tests. The Carroll analysis Glr factor also had, as expected, relatively strong loadings for the delayed recall versions of both tests (Memory for Names–Delayed Recall, MNADR = *.73*; Visual–Auditory Learning–Delayed Recall, VSAUDR = *.32*), tests which were not included in the MC analyses. Instead, in the MC analyses, relatively weaker Gl loadings were observed for the Picture Recognition (.23) and Visual Closure (.16) tests, two tests that loaded on the Carroll analysis Gv factor (Picture Recognition, PICREC = *.26*; Visual Closure, VISCLO = *.47)*. Why the difference?

One hypothesis is that the abilities measured by Picture Recognition (Gv-MV, visual memory) and Visual Closure (Gv-CS, closure speed) are considered *minor* Gv abilities (Schneider and McGrew 2018). In the MC analysis, there were two stronger indicators (Spatial Relations and Block Rotation) of one of the *major* narrow abilities of Gv (visualization) (McGrew et al. 2014; McGrew et al. 2023; Schneider and McGrew 2012, 2018), as well as a test (Planning) hypothesized to measure the Gv narrow ability of Spatial Scanning (Gv-SS).^7^ The MC analysis Gv factor may be a broader and more robust Gv factor than the Carroll analysis Gv factor. As a result, two of the Carroll Gv indicators (PICREC, VISCLO) had weak loadings on Gl in the MC analysis (Picture Recognition = .23, Visual Closure = .16). A second hypothesis, which would better explain the Carroll Glr factor, draws from Milner and Goodale’s *Two Visual Systems* (TVS) model (Goodale and Milner 2018; Milner and Goodale 2006) that specifies that visual processing abilities serve two different functional roles localized in two different clusters in the ventral and dorsal pathways or streams of the brain—visual recognition routines (i.e., vision for recognition) and real-time visual guidance (i.e., vision for action). The Picture Recognition and Visual Closure tests are non-spatial visual stimulus-based tests (i.e., vision for recognition), as are the other tests (viz., VISAUD, MEMNAM, VSAUDR, and MDADR) comprising the Carroll analysis Glr factor. In contrast, the MC analysis Gv factor is defined primarily by the clearly spatial (i.e., vision for action) Spatial Relations and Block Rotation tests.

On first inspection, four of the MC analysis factors differ from the similar factors in the Carroll analyses. However, these four MC factors are interpretable as consistent with the Carroll analysis findings. First, the tentative LANG (language) factor Carroll (2003) recommended as needing further investigation is most likely an under-identified version of the broader Grw factor in the MC analysis. In the Carroll analyses, this was a poorly defined factor that included relatively weak loadings for a test of word analysis reading skills (Grw, Word Attack; WRDATCK = *.20*) and a Gc test of general humanities information (Humanities, HUMANI = *.11*), and a strong loading for a Grw test of writing fluency or speed (Writing Fluency, WRIFLU = *.68*). In addition to the Word Attack (.58) test that was also in the Carroll analysis (WDATCK = *.20*), the MC analyses included additional Grw tests of spelling (Spelling of Sounds = .46; Spelling = .32), reading (Letter–Word Identification = .32), and writing (Editing = .22 and Writing Samples = .14). Thus, the weak language factor in the Carroll analysis is most likely an under-identified version of the robust Grw factor in the MC analysis.

Second, the Carroll analysis included a single Gs factor, while the MC analyses identified separate cognitive (Gsc) and academic (Gsa) processing speed factors. The Gsc factor in the MC analyses included salient loadings for the same Gs tests as in the Carroll analysis (Visual Matching = .68; VISMAT = *.71;* Cross Out = .57; CRSOUT = *.54*; Writing Fluency = .27; and WRIFLU = *.29*) and additional speeded or fluency tests not included in the Carroll analyses (Pair Cancellation = .79; Decision Speed = .64; Rapid Picture Naming = .43; Retrieval Fluency = .25; Reading Fluency = .38; Math Fluency = .46; and Writing Fluency = .27). The separate Gsa factor in the MC analyses was defined by three academic fluency tests (Reading Fluency = .47; Math Fluency = .37; and Writing Fluency = .25) and a test now interpreted as an indicator of Gr (naming facility narrow ability; Rapid Picture Naming = .25) (McGrew et al. 2014; Schneider and McGrew 2018). The possibility of separate cognitive and academic Gs factors is consistent with the hierarchical speed taxonomy first proposed by McGrew and Evans (2004) with a Cattell-like *g_s_* (general speed) construct at the apex. Schneider and McGrew (2018) refined the hypothesized *g_s_* hierarchy and suggested that CHC narrow speed and fluency abilities may be ordered on a continuum by their degree of cognitive complexity, as well as the possibility that cognitive and academic speed factors may represent different content facets of Gs. However, it should be noted that, in the supplementary hierarchical *g* and Horn no-*g* model analyses of these data (see Supplementary Materials Section S5, Tables S4 and S6; not interpreted in this paper, save for select salient findings bearing on Carroll’s 2003 primary research questions), the separate Gsc and Gsa factors were untenable (they displayed a negative latent factor correlation).^8^ These two factors were merged as a single Gs factor in the supplementary hierarchical *g* and Horn no-*g* analyses, and in the CHC PNA higher-stratum design analysis presented later in this paper.

The Gf factors in the two analyses are similar. In the Carroll analysis, this factor was defined by a strong loading for the Concept Formation test (CNCPTF = *.54*), a clear measure of inductive reasoning, the primary indicator of Gf and psychometric *g* (Klauer et al. 2002; Schneider and McGrew 2018). This was followed by a lower, yet salient, loading for the deductive (general sequential reasoning narrow ability) Analysis–Synthesis test (ANLSYN = *.21*) and a weak loading for a language-based Gf/Gc analogies test (Verbal Analogies, VBLANL = *.05*). In the MC analysis, Concept Formation was also the highest-loading test (.54) and Analysis–Synthesis had a lower salient loading (.18), but also had a cross-loading on the Gq factor (.20). The MC analysis Gq factor differed from the Gq factor in the Carroll analysis due to the significant Gq loadings for two quantitative reasoning (Gf-RQ) tests (Number Series = .39; Number Matrices = .38) not present in the Carroll analysis. As the Analysis–Synthesis test is designed to represent an underlying miniature math logic system, its Gf and Gq cross-loadings in the MC analyses make sense and support the MC Gf factor interpretation. Unexpected was the Understanding Directions test’s salient loading on the MC Gf factor (.40), which needs further investigation. Given that this test is a strong language-based measure of Gwm and attentional control (Schneider 2016), this finding may reflect the robust Gwm→Gf research finding (Whilhelm and Kyllonen 2021).

Finally, the Gustafsson *Gf = g* structural view of intelligence was not supported by the supplementary hierarchical *g* and Horn no-*g* analyses reported in Supplementary Materials Section S5. In the hierarchical *g* model (Table S4), the Gf factor did not have a high *g* factor loading approaching unity. Instead, the Gf factor loading on *g* (.83) was similar to that for Gl (.83) and below the higher *g* loadings for Gc (.89), Grw (.88), Gq (.88), and Gwm (.85). Accordingly, the Gf latent factor correlations in the Horn no-*g* model (Supplementary Materials Table S6) with the other eight factors were not noticeably stronger than the Grw, Gc, Gq, and Gwm factor correlations with their other eight respective CHC factors.

In summary, the combined McGrew–Carroll exploratory–confirmatory analyses of 46 WJ III tests in the age 14–19 norm sample produced robust factor results consistent with Carroll’s (2003) analyses. Carroll was pleased with these findings when we jointly interpreted the EFA-SL results during our May 2003 working session in Alaska. Based on the analyses, logic, and assumptions articulated by Carroll (2003), the collective EFA-SL/CFA findings support the Carroll 3S model of the structure of cognitive abilities over the Gustafsson Gf = *g* and Horn no-*g* models. Carroll would have been pleased with this conclusion. As stated in his own words after our joint work session in May 2003 (Carroll, personal communication, 6-11-03), “it is truly marvelous that enough data from these factors had accumulated to make their independence specifiable. Evidence now seems to be accumulating about the existence, interpretation, distribution and meaning [of] the factors G, Gf, Gc and others” (see Supplementary Materials Section S6).^9^

### 3.3. A Note on Factor Analysis as per Carroll: Art and Science

The factor analyses reported above are based on the EFA-SL methods used by Carroll in his seminal work combined with the additional CFA methods used in his final 2003 publication. Carroll’s 1993 seminal factor analysis treatise used EFA-SL methods that were available when he started his project in 1981 (Carroll 1985), which he continually improved during his decade-long program of research (Carroll 1993). Carroll started his analyses well before today’s sophisticated EFA and CFA methods proliferated and matured to their current status. Reflecting his well-known humility and ability to engage in self-critique (Carroll 1998), Carroll readily acknowledged the limitations of the methods, procedures, and software he used to produce his conclusions (Carroll 1993, 1998). His insights regarding factor analyses are illustrated in one of his comments that the value of factor analysis methods was in a *negative sense* (Anderson 1985)—“when individual differences in two variables are *uncorrelated*, or appear on different factors, I think it is an indication that the underlying processes are likely to be quite *different*, or at least dependent on quite different stimulus characteristics. I observe much more caution in inferring that the underlying processes are the *same* when individual differences on two variables show significant correlations or appear on the same factor” (Carroll 1985, p. 203, emphasis added).

I was fortunate to learn important tacit EFA and CFA knowledge during my 17 years of interactions with Carroll, and particularly my private one-to-one tutelage with Carroll in May 2003. Anyone who reads Chapter 3 (Survey and Analysis of Correlational and Factor-Analytic Research on Cognitive Abilities: Methodology) of Carroll’s 1993 book, as well as his self-critique of his seminal work (Carroll 1998) and other select method-focused post-1993 publications (Carroll 1995, 1997), should conclude what is obvious—to Carroll, factor analyses were a blend of art and science. As articulated by some of his peers (see footnote #2), his research reflected the work of an expert with broad and deep substantive knowledge of research and theories in intelligence, cognitive psychology, and factor analysis methods.

In 2003, after Carroll had been using CFA to augment his initial EFA analyses for at least a decade, Carroll expressed (to me during our May 2003 work week) that he was often concerned with the quality of some reported factor analyses (both EFA and CFA) of popular clinical IQ tests or other collections of cognitive ability measures (Carroll 1978, 1991, 1995, 2003). Carroll’s characteristic positive skepticism regarding certain reported factor analyses was first articulated (as far as I know) in the late 1970’s, when he stated “despite its many virtues, factor analysis is a very tricky technique; in some ways *it depends more on art than science*, that is, more on intuition and judgment than on formal rules of procedure. People who do factor analysis by uncritical use of programs in computer packages run the risk of making fools of themselves” (Carroll 1978, p. 91; emphasis added). It is my opinion that Carroll would still be dismayed by some of the EFA and CFA studies of intelligence tests published during the past two decades that often used narrow or restricted forms of factor analysis methods and rigid formal statistical rules for decision-making, with little attempt to integrate contemporary substantive research or theory to guide the analysis and interpretation of the results (e.g., see Decker 2021; Decker et al. 2021; McGrew et al. 2023).

Carroll’s unease was prescient of recently articulated concerns regarding two aspects of the theory crises in structural psychological research—the conflation of statistical (primarily factor analysis) models with theoretical models and the use of narrow forms of factor analysis methods (Fried 2020; McGrew et al. 2023). First, many intelligence test batteries only report CFA studies in their technical manuals. EFA results, which often produce findings that vary from CFA findings, are frequently omitted. This often leads to debates between independent researchers and test authors (or test publishers) regarding the validity of the interpretation of composite or cluster scores, leaving test users confused regarding the psychometric integrity of composite score interpretations. McGrew et al. (2023) recently recommended that intelligence test manuals, as well as research reports by independent researchers, include both EFA and CFA (viz., bifactor *g*, hierarchical *g*, and Horn no-*g* models), as well as psychometric network analysis (PNA) and possibly multidimensional scaling analyses (MDSs; McGrew et al. 2014; Meyer and Reynolds 2022). As stated by McGrew et al. (2023), “such an ecumenical approach would require researchers to present results from the major classes of IQ test structural research methods (including PNA) and clearly articulate the theoretical basis for the model(s) the author’s support. Such an approach would also gently nudge IQ test structural researchers to minimize the frequent conflation of theoretical and psychometric *g* constructs. Such multiple-methods research in test manuals and journal publications can better inform users of the strengths and limitations of IQ test interpretations based on whatever conceptualization of psychometric general intelligence (including models with no such construct) underlies each type of dimensional analysis” (p. 24).

The frequent conflation of psychometric and theoretical *g* in most intelligence test structural research resonates Carroll’s concern regarding the unbalanced professional training and research activities of some educational researchers who *lean heavily on methodology and statistics, with little attention to substantive knowledge or theory*. He described such researchers as “hard-nosed methodologists, the statistician, the test maker, the ‘measurement man’ … the model maker” (Carroll 1985, p. 384). He further opined that a highly sophisticated statistically driven research method with valid measures “is only a tool for testing good ideas; it cannot create ideas of any sort, except in so far as its *results* may suggest good ideas to the mind of the researchers. The good ideas must come from somewhere….they rarely come from people who are purely methodologists or ‘computer scientists’^10^ … the educational researcher who conforms too closely to the idea of the ‘computer scientist’ runs the risk of being written off as a pure technician, somewhat analogous to the ‘paramedical specialist’ who is ancillary to the physician” (Carroll 1985, p.384).

Wasserman (2019), in the context of CHC theories factor analysis research, recently struck a similar chord when he stated that “the good news is that in our age of rapid technological advancement, CHC has stimulated new, out-of-the-box thinking about cognitive and intellectual theories in psychological science. My hope is that some of our ‘psychometric iconoclasts’—researchers who conduct the same types of critical statistical analyses on every new edition of every new test—will turn their attention to improving CHC theory or building better tests and theories of their own. Ultimately, there is much more satisfaction in creating something new than tearing something down” (p. 263). I would also add that it is much *more difficult* to create something new than to tear something down. The expertise of ‘psychometric iconoclasts,’ who repeatedly use the same narrow class of statistical methods and rarely combine them with significant substantive knowledge and theoretical ideas, is limited.

## 4. In Search of Cognitive and Achievement Causal Mechanisms: A Carroll Higher-Stratum Psychometric Network Analysis

Most psychologists interested in intelligence are familiar with Carroll’s 1993 seminal treatise on the structure of the cognitive ability domain. However, many younger contemporary psychologists are likely unaware that Carroll also produced seminal research in psycholinguistics, the assessment of foreign language aptitude, and the postulation of his elegant *model of school learning* (Anderson 1985; Lubinski 2004). As per Anderson’s assessment, “few ideas have had a more profound influence on educational research and practice as those embedded in John Carroll’s model of school learning” (Anderson 1985, p. 3). A common thread in Carroll’s multiple research projects was a desire to understand learning, especially school learning. Readers interested in understanding his astute and comprehensive understanding of individual differences research related to school learning should read Anderson’s (1985) book tribute to Carroll’s early research—*Perspectives on School Learning: Selected Writings of John B. Carroll*.

Likely also unknown to many contemporary psychologists is Carroll’s earlier attempts (Carroll 1974, 1976, 1980, 1981) to identify and organize research regarding elementary cognitive causal mechanisms that underlie psychometrically identified cognitive abilities. As stated by Wasserman (2019),

After talking with Carroll, I began to worry that none of us really knew very much about the mental mechanisms and processes involved with 3S and Horn–Cattell broad abilities. I was floored when I later learned that Carroll (1974, 1976, 1981) had made serious efforts to characterize cognitive factors in terms of their constituent cognitive processes, and that he had developed a complete taxonomy of cognitive processes used in the performance of elementary cognitive tasks before he started his famous survey of mental abilities. Carroll’s ultimate goal, I believe, was to survey the full range of cognitive factors with each factor broken down into its constituent elementary cognitive processes. It would be analogous to setting up Linnaeus’s eighteenth-century binomial nomenclature—which introduced the standard hierarchy of class, order, genus, and species—to classify species based on their DNA!

(p. 254)

Carroll believed that many “special abilities, aptitudes, achievements, and skills [are] worthy of measurement, *whatever their relation to general intelligence might be*” (Carroll 1993, p. 36; emphasis added). Furthermore, Carroll stated that “in my view, lower-order factors (both Stratum I and II) are worthy of scientific study, not only for themselves, but also for their *possible social and practical importance*” (Carroll 1996, p. 13; emphasis added). Based on the confluence of his beliefs that some specific cognitive abilities have practical importance and his efforts to understand the basic elementary cognitive mechanisms underlying these abilities, Carroll likely would have been interested in, and most likely would have looked favorably on, the relatively new psychometric network analysis (PNA) methods to explore cognitive abilities and cognitive–achievement relations. After a brief explanation of PNA methods, a PNA of CHC factor composite variables derived from the MC analysis described earlier is presented. I believe Carroll would have viewed such analyses as an appropriate extension of much of his life’s scholarly work.

### 4.1. Psychometric Network Analysis Methods: A Brief Introduction

Factor analysis methods have been the descriptive taxonomic workhorse of 20th century cognitive ability research. These methods are based on the premise that, among a collection of positively correlated cognitive ability tests (i.e., the *positive manifold*), there is a “common cause” latent variable “out there” that can be “detected” (van der Maas et al. 2014). A significant limitation of common cause models is that, regardless of how psychometric *g* is specified (e.g., bifactor *g* vs hierarchical *g*), psychometric *g* is explicitly operationalized as the primary cognitive mechanism or lever for producing change, either directly on the abilities measured by the manifest test indicators (bifactor *g* model) or on the stratum II broad CHC constructs (hierarchical *g*). This can be seen in factor analysis path diagram figures, where the single-headed arrows originate from the psychometric *g* factor (typically an oval) to either the manifest indicators (i.e., tests represented by rectangles) or first-order factors (ovals). The origin and direction of the arrows imply that *g* is the primary causal mechanism that explains performance on individual cognitive tests, either directly or as mediated through the first-order CHC factors. A model with such a central dominant psychometric *g* factor “bars complex interactions both within the construct of intelligence itself and with its adjacent systems” (Savi et al. 2021, p. 5).

In contrast, modern network-based models of intelligence (e.g., process overlap theory and dynamic mutualism) (Protzko and Colom 2021; van der Maas et al. 2006, 2019) disregard the assumption that latent unobserved factor constructs cause the positive manifold among cognitive ability tests. Instead, network models (and psychometric network analysis methods) are grounded on the assumption that cognitive tests positively covary because they are *the by-product (an emergent property) of a complex system of biological and cognitive mechanisms* (Hampshire et al. 2012; van der Maas et al. 2019). That is, modern network cognitive ability theories posit that psychometric *g* is the result of, and not the cause of, the positive manifold between tests in an IQ test battery (Conway et al. 2021; Fried 2020; Kovacs and Conway 2016, 2019; Hampshire et al. 2012; Kan et al. 2019). While common cause factor analysis models have dominated 20th-century intelligence research, network theories and methods “will dominate the twenty first [century of human intelligence research]” (Savi et al. 2021, p. 1). Network theories and methods can move intelligence research forward by going beyond the descriptive function of developing cognitive taxonomies (e.g., CHC theories) to the development and testing of theories and models that explicate possible underlying cognitive causal mechanisms and, in turn, inform possible interventions (McGrew et al. 2023).

**Figure 1:**
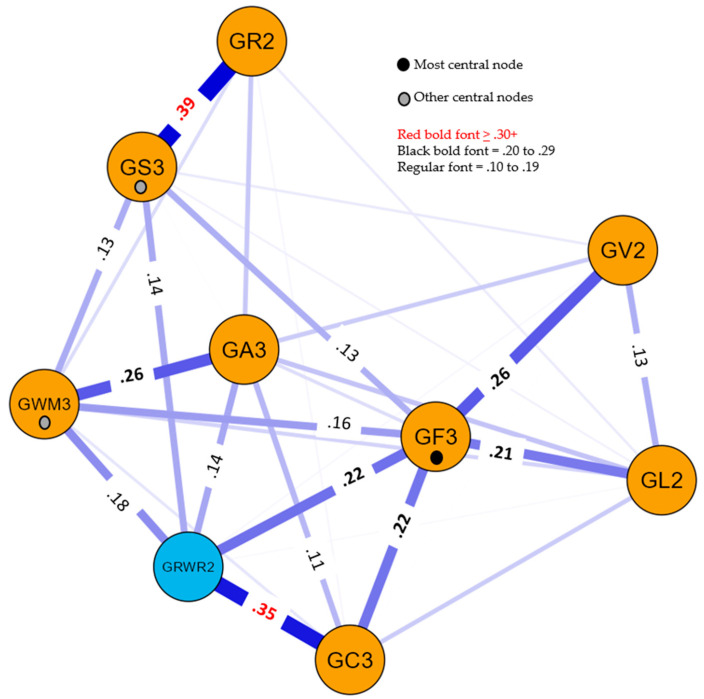
Weighted undirected network structure of eight WJ III CHC cognitive composite measures and one reading (GRWR2) composite measure in the cognitive–reading network model. *Note.* Numbers are the edge weights greater than or equal to .10 (see key for three-category system of relative weight size). The three most central nodes are designated by black and gray circles on nodes (see manuscript text).

**Figure 2:**
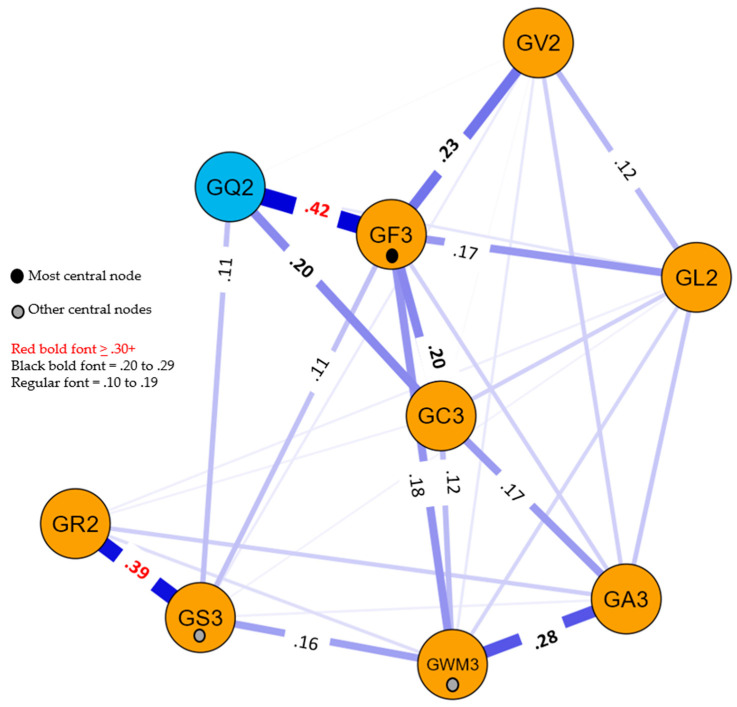
Weighted undirected network structure of eight WJ III CHC cognitive composite measures and one math (GQ2) composite measure in the cognitive–math network model. *Note.* Numbers are the edge weights greater than or equal to .10 (see key for three-category system of relative weight size). The three most central nodes are designated by black and gray circles on nodes (see manuscript text).

Atheoretical, data-driven, and descriptive PNA models can illuminate previously hidden insights by examining high-dimensional data where tests can concurrently serve as both predictor and predicted variables (Angelelli et al. 2021; Epskamp et al. 2017). Briefly, in PNA, the manifest measures (tests) are represented as *nodes*. Links between nodes (*edges*) are identified and the strength of the non-directional relations between each pair of nodes are statistically estimated (partial correlation coefficients). Thicker edges represent stronger node associations. In the network model, all patterns of pairwise conditional test relations are statistically estimated independently of relations with all other tests in the network. All salient node relations are presented in a multidimensional visual–graphic network (see Figure 1 and Figure 2). PNA models can also identify groupings or communities of measure nodes akin to latent variable common cause factors (Golino and Epskamp 2017). The topography of a cognitive abilities test network is characterized by tools from network science (i.e., *centrality* metrics) (Borsboom et al. 2021; Bulut et al. 2021; Jones et al. 2018; Neal and Neal 2021; Robinaugh et al. 2016). These metrics include the *closeness* index (how close a measure node is, on average, to all other measure nodes in the network), the *betweenness* index (how frequently a measure node lies on the shortest path connecting any two other nodes in the network), and the *strength* index (how strongly, on average, a specific measure node is connected to all other nodes in the network.

The benefits of PNA (over common cause factor models) are multiple (Angelelli et al. 2021; Borsboom et al. 2021; Epskamp et al. 2017; McGrew et al. 2023). In the current context, “the primary value of these descriptive models is their ability to function as a bridge to theory formation and the ability to hypothesize, and empirically test or statistically simulate, potential causal mechanisms in the network (Borsboom et al. 2021; Haslbeck et al. 2021)” (McGrew et al. 2023, p. 5). PNA models can be used to generate causal hypotheses between abilities measured by individual node measures, offering insights regarding the most likely influential targets (or target systems) for intervention (Haslbeck et al. 2021; McGrew et al. 2023).

As noted by Protzko and Colom (2021), PNA is conceptually akin to bifactor factor analysis models that first remove the psychometric *g* variance in a collection of cognitive tests before examining the residual variance that may represent common broad CHC dimensions. PNA accomplishes the same effect by analyzing the partial correlation matrix of all tests, where, for each pair of tests, the variance shared with all other measures (i.e., psychometric *g*) in the network is statistically removed (McGrew et al. 2023); “this is not to be confused with the assumption that psychometric *g* is essential to PNA research” (p. 17). In the current context, the PNA results presented below represent the relations between specific broad CHC ability measures after psychometric *g* has been removed—a model consistent with Carroll’s strong *g* position alongside his belief that some specific broad and narrow abilities are important in applied settings.

### 4.2. A Carroll-Inspired Higher-Stratum PNA Analysis of Cognitive–Achievement Variables

#### 4.2.1. Methodology

The PNA of all 46 WJ III tests from the MC analysis would have produced PNA network models of substantial complexity that would defy easy interpretation. More importantly, Carroll (1993, 1995) recommended that cognitive ability research needed more higher-stratum research designs where composite variables are established to represent stratum II broad CHC abilities. According to Carroll (1993), *higher-stratum* designs are where “variables are sums or averages of scores obtained on two or more tests of a given first-stratum factor” (p. 579). Great care and caution must be used to best represent the broad CHC factor (i.e., adequate construct representation). Thus, the results from the final MC analysis reported in Table 1 were used to create eight two-to-three-test composite variables representing nine of the reported first-order CHC broad factors in Table 1.

The WJ III *W*-score, which is a direct transformation of the Rasch logit scale (with a center of 500 points at the age of 10 years) (McGrew and Woodcock 2001; McGrew et al. 2014), was the selected metric for the creation of the CHC composite variables. The *W*-score is an equal interval growth score strongly influenced by developmental (age) variance. PNA methods, for each pair of measures, statistically remove the variance shared with all other measures (including the *W*-score’s developmental variance) before constructing the network.

For the reasons reported earlier, only a single Gs composite was created (and not separate Gsc and Gsa composites). The final set of composite variables, where each CHC ability abbreviation is followed by a “2” or “3” (designating the number of test scores summed and averaged to create the composite), were Gc3 (comprehension–knowledge; Verbal Comprehension, General Information, and Oral Comprehension), Gf3 (fluid reasoning; Concept Formation, Analysis–Synthesis, and Number Series), Gs3 (processing speed; Visual Matching, Cross Out, and Pair Cancellation), Ga3 (auditory processing; Sound Blending, Incomplete Words, and Sound Patterns–Voice), Gwm3 (working memory; Auditory Working Memory, Memory for Words, and Numbers Reversed), Gv2 (visual–spatial processing; Spatial Relations and Block Rotation), Gl (learning efficiency; Visual–Auditory Learning and Memory for Names), Gr2 (retrieval fluency; Retrieval Fluency and Rapid Picture Naming), Gq2 (quantitative knowledge; Applied Problems and Calculation), and Grwr2 (reading/writing—reading; Letter–Word Identification and Passage Comprehension). Given that not all subjects had scores for all 46 tests, the sample size for the PNA analyses of composite variables was smaller than that used for the factor analyses reported in Table 1. The listwise deletion of missing data option was used, resulting in a final data file of 670 subjects. This sample had a mean age of 16.1 years (*SD* = 1.6 years).

The analysis was completed with the open-source *JASP* network analysis software program module. Using the *JASP* default parameters, the module generated Gaussian graphical models (GGM) with the *EBICglasso* estimator (Epskamp and Fried 2018; Friedman et al. 2008). The *LASSO regularization* technique was used to estimate the edge weights (partial correlations). This statistical procedure penalizes model complexity in favor of parsimony (Epskamp and Fried 2018). The LASSO technique removes non-significant edges by estimating them to be zero. The remaining weights are typically referred to as *non-zero* weights.

Two network models were estimated, both including the Gc3, Gf3, Gs3, Ga3 Gwm3, Gv2, Gl2, and Gr2 cognitive composite variables. The reading PNA model also included the Grwr2 composite achievement variable, while the math PNA model included the Gq2 composite achievement variable. The separate achievement domain models were analyzed to determine whether the CHC cognitive composite variables demonstrated achievement domain-general or domain-specific relations between cognitive variables and reading and math achievement. The PNA *centrality* indices for both models are presented in Supplementary Materials Section S7, Table S9. Centrality indices are often presented in the form of standardized *z*-scores. In this study, the *relative* centrality *z*-scores were rescaled so that the strongest test had a value of 1.0 and the weakest had a value of 0. As demonstrated by McGrew et al. (2023), this rescaling does not impact interpretation. The relative centrality metrics are analogous to common psychometric test characteristic information (e.g., *g*-loadings, factor loadings, reliabilities, etc.) and, thus, are more familiar to intelligence test researchers. In the evaluation of the relative size of the centrality metrics, the values for the two achievement composites (Grwr2 and Gq2) were excluded. The resultant reading and math PNA models are presented in Figure 1 and Figure 2.

#### 4.2.2. Results and Discussion

In both models, the cognitive variables are designated by the yellow nodes and the respective achievement domain is represented by a blue node. The strength of the relations (*edge* weights; partial correlation coefficients) between each pair of measures is represented by the width of the connecting lines in the visual network models. In the reading model, 32 of the 36 edge weights were non-zero (sparsity index = .11), while in the math model, 30 of the 36 edge weights were non-zero (sparsity index = .17). The relatively large number of non-zero network edges is likely due to the high power of the statistical analyses due to the relatively large sample size. Only the significant edge weights that were greater than .10 are included in both figures, differentiated by the magnitude of the weights (.10 to .19, .20 to .29, ≥ .30). The complete weights matrix tables are included in Supplementary Materials Section S7, Table S10.

The three most central nodes in each model were the same. The Gf3 variable was consistently the most central node, as indicated by relative centrality strength, closeness, and betweenness indices of 1.0 in both models. In the reading model, the Gwm3 measure was the second highest variable as per the strength (.75) and closeness (.83) indices, while Gs3 was third in strength (.68) and the second highest in the betweenness index (.60). Similarly, in the math model, the Gwm3 measure was the second highest variable as per the strength (.65) and closeness (.86) indices, while Gs3 was third in strength (.61) and was the second highest in the betweenness index (.55). The centrality of these three composite measures (Gf3, Gwm3, and Gs3) is designated by the small black or gray circles on their respective nodes in Figure 1 and Figure 2.

Currently, both models are descriptive, not explanatory. For example, the .39 weight between Gr2 and Gs3 in both Figure 1 and Figure 2 may suggest that Gr2 has a *unidirectional* effect on Gs3 (or vice versa) or the relationship is *bidirectional*. The triangle formed by the significant Gf3, Gv2, and Gl2 links in both figures may suggest a *common cause* (Cattell’s *g_f_* as an overarching cause of all three) or a *common effect* (Gv2 and Gl2 are correlated, and both exert a common effect on Gf3 (Borsboom et al. 2021)). The primary value of these descriptive models is their ability to function as a bridge to theory formation and the ability to hypothesize and empirically test or statistically simulate potential causal mechanisms in the network (Borsboom et al. 2021; Epskamp et al. 2017; Haslbeck et al. 2021; McGrew et al. 2023). The ability to bridge the descriptive networks to theories and causal mechanisms requires *considerable substantive knowledge*. Furthermore, moving from simple exploratory descriptive models to formal theory and, recursively, from theory-implied data models to the refinement of formal theories is complex (Epskamp et al. 2017; Haslbeck et al. 2021; Lunansky et al. 2022) and beyond the scope of this paper.

Few, if any, validated theoretical causal mechanisms between and among *all* major CHC abilities exist in the literature. However, Hajovsky et al. (2022) recently reported, via exploratory meta-structural equation modeling applied to data from multiple cognitive and achievement batteries, how three established theories of cognitive and achievement development (i.e., the developmental cascade theory, Cattell’s investment theory, and an expanded simple view of the reading model) could be applied to a complex interconnected network of CHC constructs and measures. Given that the purpose of the interpretation of Figure 1 and Figure 2 is to demonstrate the potential of Carroll’s higher-stratum design cognitive–achievement PNA models, only the two most recognized theories (i.e., developmental cascade and Cattell’s investment theory) were applied to the two models.

Briefly, the concept of developmental cascades has a long history in developmental psychology. *Developmental cascades* “refer to the cumulative consequences for development of the many interactions and transactions occurring in developing systems that result in spreading effects across levels, among domains at the same level, and across different systems or generations. Theoretically these effects may be direct and unidirectional, direct and bidirectional, or indirect through various pathways, but the consequences are not transient: developmental cascades alter the course of development” (Masten and Cicchetti 2010, p. 491). In the cognitive abilities literature, robust research has established the general *age (maturation)—> Gs—> Gwm—> Gf—> acquired knowledge* (e.g., Gc and achievement domains) developmental cascade theory (De Alwis et al. 2014; Fry and Hale 1996, 2000; Kail 2007; Kail et al. 2016; McGrew 2005), although more complex and developmentally nuanced versions have also been reported (Demetriou et al. 2014; Tourva and Spanoudis 2020). As per Cattell’s *investment theory* (Cattell 1971, 1987), the consistent application of fluid reasoning (*g_f_*) to learning via schooling and experience generates new crystallized acquired knowledge (*g_c_*) (Schneider and McGrew 2018). The general mechanisms of this investment come from societal (e.g., educational resources), familial (e.g., resources and expectations), and personal (e.g., personality, motivation, and volition) investment mechanisms (McGrew 2022; Schneider and McGrew 2018).

**Figure 3:**
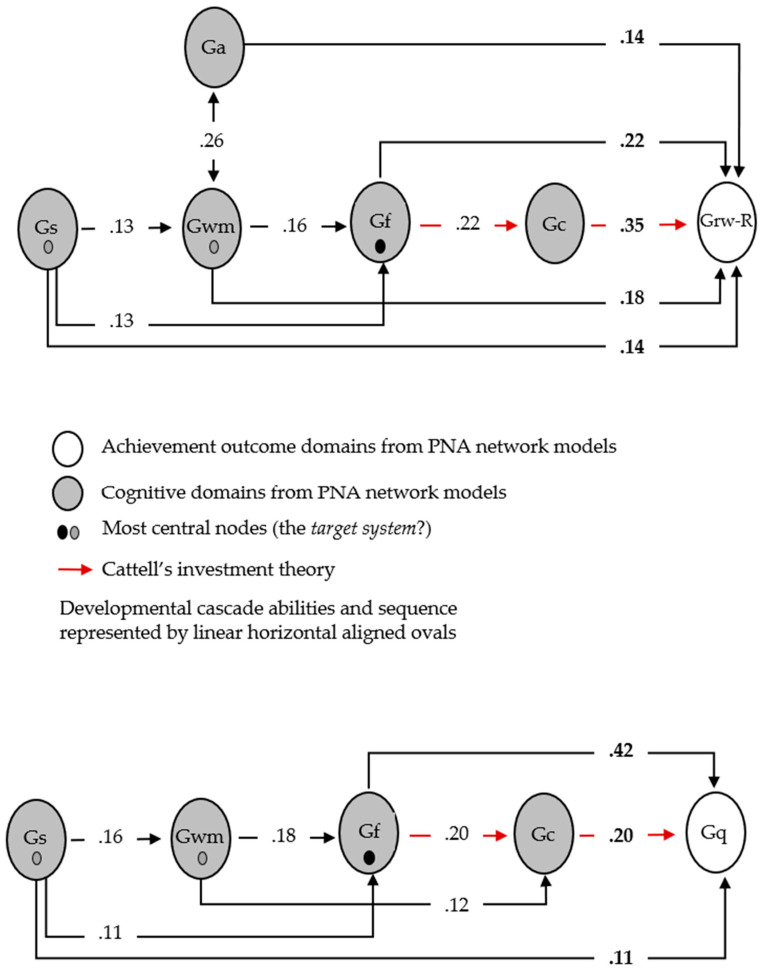
CHC cognitive, reading, and math composite variables (and weights) from Figure 1 and Figure 2 as organized as per the developmental cascade and Cattell investment theories. *Note*. Bold font indicates direct cognitive path links to achievement composite measures.

Figure 3 represents a first, albeit simplistic, attempt to identify the developmental cascade and Cattell investment mechanism relations and results embedded in the reading and math PNA model figures. Two assumptions simplified the generation of these hypothesized models. First, it was assumed that the reading (Grw-R) and math (Gq) variables were “downstream” achievement outcomes reflecting the cumulative impact of “upstream” cognitive variables. Second, the order of the cognitive variables in the developmental cascade sequence was unidirectional from left to right (likely an oversimplification of reality). Third, the developmental cascade and Cattell investment theories overlapped in a portion of the sequence with Gf preceding the development of acquired knowledge (Gc, Grw-R, and Gq). An inspection of Figure 3 suggests the following general conclusions.

First, the strength of the links between the central developmental cascade cognitive variables (Gs, Gwm, Gf, and Gc) is similar in both models. This suggests that most of the relations between CHC cognitive variables are similar and do not vary when different achievement variables are added. Second, the reading model (but not the math model) includes the Ga domain, a finding consistent with the extant CHC reading literature that suggests that Ga may be a reading domain-specific cognitive ability. In the reading model Ga has a direct link to reading (.14), but also a possible indirect mediated link via Gc (.11). The same Ga→Gc link (.17) is present in the math model, but there is no direct link from Ga to math; thus, Ga is not included in the math model in Figure 3. The link of Ga to language (Gc) is consistent with research that has demonstrated that Ga may serve an important scaffolding function for the development of cognition and language (Conway et al. 2009; Schneider and McGrew 2018). Finally, the number of cognitive domains with direct links (edge weights) with achievement is greater for reading (five abilities; Gs, Gwm, Gf, Gc, and Ga) than for math (three abilities; Gs, Gf, and Gc). This suggests that reading is a more cognitively complex skill than math at ages 14 through 19. Both Gf and Gs displayed links with reading (.22 and .14, respectively) and math (.42 and .11, respectively). The Gf link is considerably stronger for math (.42) than reading (.22), suggesting that Gf may be more important for math (more math domain-specific) than reading. A major source of the stronger Gf–math achievement (Gq) link is likely due to two of the three Gf3 composite variables being a miniature math logic system task to assess deductive reasoning (i.e., Analysis–Synthesis) or a classic measure of quantitative reasoning (Number Series; Carroll 1993). Conversely, the Gc link is stronger for reading (.35) than math (.20), which suggests that verbal knowledge and comprehension abilities are relatively more important for reading when compared with math at ages 14 through 19. The differential reading (Ga and Gc) and math (Gf) findings are consistent with the extant research on cognitive–achievement relations that has used multiple regression (no-*g*) or hierarchical *g* SEM models (McGrew and Wendling 2010). A notable difference between the models is that, in the reading model, Gwm has a direct link to reading (.18), but not Gq. In the math model, Gwm instead has a direct link (.12) to Gc.

The remaining portions of the two models are, currently, only open to general speculation and need further investigation. The strong Gr2–Gs3 link (.39 in both Figure 1 and Figure 2) could be interpreted as the hierarchical *g_s_* proposed by Schneider and McGrew (2018) or, conversely, might suggest that Gs is a causal ability for Gr (Forthmann et al. 2019). As mentioned previously, the Gf3, Gv2, and Gl2 triangle in both figures could suggest unknown unidirectional or bidirectional relations or a common cause (e.g., *g_f_*) underlying the three constructs.

The .26 (reading; Figure 1) and .28 (math; Figure 2) Ga3–Gwm3 link is intriguing and warrants more comment. In the reading model in Figure 3, this path is designated by a bidirectional arrow that, in part, reflects uncertainty in the Gwm and Ga causal relations. Horn (1985), in an early developmental and information processing cognitive hierarchy, placed “broad auditory thinking” (Ga) downstream from “short-term acquisition retrieval” (SAR; i.e., SAR—>Ga). However, Horn’s SAR reflected a component of Gwm, namely short-term memory storage. It is likely that Horn’s (1985) model would have been different if specified after contemporary notions of working memory (Gwm) became available. Hajovsky et al.’s (2022) exploratory meta-structural equation modeling-based model reported a similar Gwm→Ga causal direction. However, if Ga is conceptualized and measured as more of a working memory subordinate system “limited to the storage of heard and spoken speech” (Baddeley 2012, p. 12), Ga might be conceptualized more as reflecting the phonological loop (Ga) working within the context of working memory (Gwm) (Baddeley 2012), which might be represented by bidirectional arrows. However, Baddeley (2012) also acknowledged that the phonological loop (Ga) might also “provide a means of action control” (p. 11) (e.g., internal verbal self-instruction that influences working memory), which would be consistent with a Ga→Gwm causal direction. Another possibility may be that Gwm and Ga reflect a common cause serial or sequential processing cognitive dimension (Kaufman and Kaufman 1983; Naglieri and Das 1997), requiring a bidirectional arrow that reflects their shared covariance. Finally, if Ga is conceptualized as delivering only a fuzzy trace signal, then the phonological loop hypothesis is flawed and perhaps Ga→Gwm is the correct causal direction. Confounding the possible Gwm and Ga relationship is how the two constructs are measured. Most contemporary Gwm tasks minimize Ga involvement, while, in contrast, most Ga tasks fail to minimize working memory demands. Additional research with clearly operationally defined Gwm and Ga measures, especially in time-series longitudinal studies, is needed to solve the Gwm–Ga “chicken or the egg” question.

The three central nodes designated in Figure 1 and Figure 2 are also included in Figure 3. Interestingly, the Gs, Gsm, and Gf trilogy is a central core of the cognitive developmental cascade theory. This Gs—>Gwm—> Gf trio could represent what Haslbeck et al. (2021) defined as a possible *target system* of the CHC network—“the particular parts of the real world and the relationships among them that give rise to the phenomena of interest … theories can thus be understood as models that represent the target system” (p. 2). In a related analysis using the WJ IV norm data, McGrew et al. (2023) reported a CHC PNA of carefully selected CHC construct measures that identified the *working memory–attentional control (Gwm-AC) complex* (comprised of Gs, Gwm, and Gr cognitive efficiency constructs) as the most likely target system for intellectual functioning.

Traditional statistical prediction models of achievement, such as multiple regression, provide few clues regarding potential complex causal relations between and among variables. The PNA cognitive–achievement interpretations offered here, although speculative, when informed by the extant substantive research and theoretical literature, have greater potential to elucidate the complex relations between and among CHC cognitive and achievement constructs. The descriptive PNA models (Figure 1 and Figure 2) can be “explored with various tools from network science (e.g., exploratory and confirmatory PNA; exploratory stepwise search algorithms to guide the removal or addition of nodes to improve the model; *in silico* mathematical simulations where changes in network nodes are statistically modified [or constrained] to see how the effect propagates through the entire network—and potentially reveals causal mechanisms in the network; etc.) (Epskamp et al. 2017; Haslbeck et al. 2021; Lunansky et al. 2022)” (McGrew et al. 2023, p. 6).

PNA could assume a pivotal role in improving CHC cognitive–achievement relations SEM modeling research as it “acts as a natural interface between correlation and causality … [as] the typical attempt to determine directed SEMs from correlation structures in fact appears somewhat haphazard in psychology, a historical accident in a field that has been prematurely directed to hypothesis testing at the expense of systematic exploration” (Epskamp et al. 2017, pp. 924–25). PNA methods could facilitate CHC SEM modeling via the systematic identification of relations between multiple variables unfettered by concerns for “direct causal relations, reciprocal causation, latent common causes, semantic overlap between items [variables], or homeostatic coupling of parameters” (Epskamp et al. 2017, p. 925).

Psychometric network analysis of CHC-derived higher-stratum composite variables, augmented by substantive theoretical knowledge and tools from network science, can serve as a new lens to identify and understand relations between cognitive abilities and to potentially improve intellectual functioning and school learning, which are all topics near and dear to Carroll’s enduring legacy.

## 5. Summary

Approximately 30 years ago, John “Jack” Carroll published, arguably, one of the most paradigm-shifting research syntheses in the field of intelligence. Carroll was the right researcher, at the right time, with the right degree of expertise regarding individual differences in cognitive abilities to provide the field of intelligence its first empirically grounded, defensible, theory-based taxonomy of human cognitive abilities. Carroll’s distillation of a century of psychometric research that started in earnest in the early 1900′s with Spearman was in the form of Carroll’s three-stratum (3S) theory of cognitive abilities. Few others could have produced such a magnum opus regarding the structure of human cognitive abilities (Lubinski 2004), a decade-long “retirement” project of Carroll’s (Carroll 1993).

For the latter half of the 20th century, the foundation of clinical intelligence test interpretation resembled a duct-taped mash-up of select ad hoc empirical considerations, the retrofitting of old theories to new tests, and clinical lore (Schneider and Flanagan 2015). As the result of the (initially) serendipitous and subsequent ongoing consultation between Carroll, Horn, and the Woodcock–Johnson (WJ, WJ-R, and WJ III) author teams, Carroll’s 3S theory was commingled with Raymond Cattell’s and John Horn’s Gf-Gc theory in an effort to narrow the long-standing theory–research–practice gap in applied intelligence test development and interpretation. The palpable thirst for a defensible cognitive abilities nomenclature for intelligence testing was, over a relatively short period of time, quenched by the arrival (and practical translation) of Carroll’s 3S theory. Subsequently, practical applied test development needs resulted in the informal melding of the psychometric theories of Carroll, Cattell, and Horn as the tripartite Cattell–Horn–Carroll (CHC) theory. CHC theory rapidly became the standard nomenclature for communication in the intelligence research and applied testing communities. The narrowing of the theory–research–practice gap, which was largely kick-started by Carroll’s 1993 tome, is one of Carroll’s many enduring legacies.

Not long after the arranged marriage of Cattell’s, Carroll’s, and Horn’s respective theories as CHC theory, and unbeknownst to many engaged in the infusion of CHC theory into clinical practice, both Horn and Carroll became vexed with the popularity of the singular CHC term. CHC theory gave the false impression that Cattell, Horn, and Carroll had resolved their theoretical differences. They had not. Carroll and Horn had a long-standing sharp, and often contentious, difference of opinion regarding the need for a psychometric *g* factor in a structural model of intelligence. A brief historical review of the origins and evolution of CHC theory suggests that Cattell, Horn, and Carroll’s respective psychometric theories of intelligence should now be recognized on their own merits and henceforth collectively be referred to as a family of obliquely correlated CHC theories. Of the three theories, Cattell’s Triadic theory was the greatest casualty of the success of the singular CHC theory umbrella term and should be resurrected and empirically compared with the structural models of Horn and Carroll.

In Carroll’s honor, this paper presented a previously unpublished exploratory factor analysis completed by Carroll (collaboratively with this author a little more than a month before his passing in 2003) of a large set of 46 cognitive and achievement tests. The results validated Carroll’s (2003) conclusions that his 3S structural model (i.e., *standard multifactorial view*) was a more accurate description of the structure of human cognitive abilities than the alternative Gustafsson *Gf = g* (i.e., *limited structural analysis view*) and Horn no-*g* (*second-stratum multiplicity view*) models. These findings were reinforced by the recent CFA bifactor analyses of the same 46-test dataset as reported in this paper. The EFA and CFA results collectively suggest that Carroll’s (1993) 3S structure, at the global level, still holds (Whilhelm and Kyllonen 2021). The combined EFA and CFA of 46 tests, completed as per Carroll’s factor analysis methodology, supported a model with nine broad CHC factors (i.e., Gf, Gc, Gv, Gl, Gr, Gwm, Gs, Gq, and Grw) and a dominant psychometric *g* factor at the apex. As summarized by Whilhelm and Kyllonen (2021), “there may be alternative explanations besides the three-stratum model … but for now, the positing of a set of discrete abilities whose identities are established stands” (p. 2). Carroll’s (1993) 3S model now serves as a critical reference point for future research focused on understanding the structure and causal mechanisms of human intelligence.

Carroll’s (1993) work is the capstone event of the 20th century of psychometric intelligence research, signaling the end of major discoveries regarding the structural organization of the most studied broad cognitive ability domains. However, Carroll (1993) made it abundantly clear that his synthesis was only a snapshot, and that “the picture is far from complete … many factors remain inadequately specified, and many aspects of the three-stratum theory need to be tested and refined” (p. 688). Schneider and McGrew (2012, 2018) twice updated the CHC taxonomy based on research published after Carroll’s (1993) work, often following nascent clues offered by Carroll. However, additional factor analysis studies are still needed.

First, efforts should continue to identify and clarify the number and organization of the poorly understood, or yet-to-be-identified, narrow stratum I abilities (Schneider and McGrew 2018; Wasserman 2019). Second, additional structural research is needed to better map and understand the relatively new, or less-researched, broad CHC domains of emotional intelligence (Gei), quantitative knowledge (Gq), olfactory abilities (Go), tactile abilities (Gh), kinesthetic abilities (Gk), psychomotor abilities (Gp), and psychomotor speed (Gps). Third, as per Carroll’s (1993, 1995) recommendation, research is needed to identify possible intermediate stratum cognitive ability or processing dimensions that lie between broad CHC abilities and psychometric *g* (Schneider and McGrew 2018). For example, McGrew et al. (2023), using psychometric network analysis (PNA) and related methods with CHC theory-consistent test indicators, identified possible System I (i.e., more automatic or automatized cognitive functions) and System II (more controlled deliberate cognitive functions) CHC cognitive processing dimensions (Barrouillet 2011; De Neys and Pennycook 2019; Kahneman 2011). These researchers also identified a Cattell-like *g_f_–g_c_* (Cattell 1987) CHC continuum (like Ackerman’s *intelligence-as-process and intelligence-as-knowledge* distinction; Ackerman 1996, 2018). Both the *g_f_–g_c_* and System I–II intermediate stratum dimensions may be necessary to better understand the relations and possible causal mechanisms between and among broad and narrow cognitive abilities, as well as to inform intelligence test development and interpretation practices. Whilhelm and Kyllonen (2021) have provided additional research recommendations building on Carroll’s (1993) work.

In the spirit of Carroll’s career-long search to better understand the structure and causal mechanisms underlying cognitive abilities and school learning (Anderson 1985; Wasserman 2019), and his recommendation (Carroll 1993) for more higher-stratum research designs, the EFA and CFA structural results reported here were extended by the application of the psychometric network analysis (PNA) of broad CHC cognitive and achievement composite measures. The reported CHC-based PNA models produced findings consistent with the achievement domain-general developmental cascade and Cattell investment theories of cognitive development, as well as the extant reading and math domain-specific CHC cognitive ability research. The PNA methodology and results demonstrate how this relatively new methodology can complement factor analysis by providing a framework for identifying and empirically evaluating cognitive–achievement causal relations and mechanisms, with an eye toward improved cognitive intervention research and theory formation.

It is believed that Carroll would welcome the integration of factor analysis and PNA methods. Ironically, the PNA of Carroll’s last published *g*-dominated factor analyses (Carroll 2003), and the results presented in this paper, eschew the inclusion of a psychometric *g* factor, a factor central to Carroll’s 3S model. The non-*g* PNA models would likely be viewed favorably by Carroll as they are consistent with his long-held belief that some broad and narrow cognitive abilities are central to understanding the causal mechanisms of intelligence and school learning beyond general intelligence (*g*) (Carroll 1993, 1998). Carroll was clearly one of the most eminent maestros who orchestrated the astute integration of the extant intelligence theoretical and research literature with the art and science of factor analysis methodology to produce the classic capstone masterpiece of the 20th century. PNA models are likely to be more pivotal in future studies focused on investigating the possible causal models of intelligence. The higher-stratum designed cognitive–achievement PNA research presented in this paper is a logical extension of Carroll’s legacy that can bridge the 20th and 21st centuries of psychometric intelligence research.

## Figures and Tables

**Table 1:** Bifactor CFA of WJ III 46 Tests Based on Carroll Exploratory Factor Analysis. *Note.* TM CHC = CHC classifications from WJ III technical manual. Italic font indicates different CHC factor classification based on the analysis. The 1st-order factor names are as per Schneider and McGrew (2018). O1 and O2 designate 1st- and 2nd-order factors as per Carroll’s EFA-SL software (see Supplementary Materials Section S3. Bold designates test *g* loadings of .70 or above. ^1^ Original WJ III Glr classification changed to Gl or Gr and Gsm changed to Gwm as per Schneider and McGrew (2018). ^2^ Gsc = processing speed–cognitive; Gsa = processing speed–achievement. ^3^ Number Series and Number Matrices subtests were combined as a single Numerical Reasoning test in the WJ III.

WJ III Test Name						Factors							
	TM CHC	O2 *g*	O1 Grw	O1 Gc	O1 Gl ^1^	O1 Gsc ^2^	O1 Gf		O1 Gsa ^2^	O1 Gwm ^1^	O1 Gq	O1 Gv	O1 Ga
Word Attack	Grw	0.62	0.58										
Spelling of Sounds	Grw	**0.70**	0.46										0.30
Spelling	Grw	0.65	0.32										
Letter–Word Identification	Grw	**0.74**	0.32										
Editing	Grw	**0.70**	0.22										
Writing Samples	Grw	0.67	0.14										
Picture Vocabulary	Gc	0.65		0.65									
Verbal Comprehension	Gc	**0.86**		0.45									
General Information	Gc	**0.82**		0.34									
Academic Knowledge	Gc	**0.83**		0.26									
Reading Vocabulary	*Grw*	**0.81**		0.20									
Story Recall	*Gl*	**0.82**		0.14									
Oral Comprehension	Gc	0.66		0.14									
Passage Comprehension	*Grw*	0.68		0.08									
Memory for Names	Gl	0.57			0.48								
Visual–Auditory Learning	Gl	**0.70**			0.44								
Picture Recognition	*Gv*	0.44			0.23								
Visual Closure	*Gv*	0.25			0.16								
Pair Cancellation	Gs	0.42				0.79							
Visual Matching	Gs	0.48				0.68							
Decision Speed	Gs	0.39				0.64							
Cross Out	Gs	0.51				0.57							
Retrieval Fluency	*Gr*	0.54				0.25							
Concept Formation	Gf	**0.73**					0.54						
Understanding Directions	*Gwm*	**0.81**				0.22	0.40						
Reading Fluency	*Grw*	0.66				0.38			0.47				
Math Fluency	*Gq*	0.50				0.46			0.37		0.33		
Rapid Picture Naming	*Gr*	0.43				0.43			0.25				
Writing Fluency	*Grw*	0.58				0.27			0.25				
Memory for Words	Gwm	0.57								0.50			
Numbers Reversed	Gwm	0.58								0.40			
Memory for Sentences	Gwm	0.66		0.14						0.37			
Auditory Working Memory	Gwm	**0.70**								0.37			
Calculation	Gq	0.59									0.42		
Number Series ^3^	Gf	**0.73**									0.39		
Number Matrices ^3^	Gf	**0.72**									0.38		
Applied Problems	Gq	**0.76**									0.34		
Analysis-Synthesis	Gf	**0.73**					0.18				0.20		
Spatial Relations	Gv	0.50										0.52	
Block Rotation	Gv	0.49										0.39	
Planning	Gv	0.38										0.29	
Sound Blending	Ga	0.56											0.58
Auditory Attention	Ga	0.42				0.23							0.41
Incomplete Words	Ga	0.48											0.34
Sound Patterns–Voice	Ga	0.49											0.20
Sound Awareness	Ga	**0.83**											

## Data Availability

Data used in this study are the proprietary property of Riverside Insights (formerly, Riverside Publishing). All data are solely owned and licensed by Riverside Insights and thus cannot be shared by the authors in any form or format. Requests to access the data should be directed to Riverside Insights.

## Notes

1 Given the repeated reference to Carroll’s (1993) book and the 3S theory included therein, hereafter, in this paper, this work is referred to as Carroll’s book or Carroll’s work.

2 The broad CHC ability names, definitions, and abbreviations are drawn from Schneider and McGrew (2018): Gc (Comprehension-Knowledge), Grw (Reading and Writing), Gq (Quantitative Knowledge), Gf (Fluid Reasoning), Gwm (Short-term Working Memory), Gv (Visual–spatial Processing), Ga (Auditory Processing), Gl (Learning Efficiency), Gr (Retrieval Fluency), and Gs (Processing Speed). Definitions for these broad and narrow CHC abilities are included in Supplementary Materials Section S1.

3 See the personal reflections of Julian Stanley, David Lubinski, A. T. Panter, and Lyle V. Jones published by the *Association for Psychological Science* (APS) on 9 November 2003 (https://www.psychologicalscience.org/observer/in-appreciation-john-b-carroll (accessed on 4 October 2021)).

4 The lower-case italic subscripted notation (e.g., *g_f_* and *g_c_*) recognizes that Cattell’s *general capacities* were more consistent with (but different from) Spearman’s general intelligence (*g*). The more common upper case CHC theory ability notation (e.g., Gf and Gc) acknowledges both Horn and Carroll’s notions of *broad* abilities. See Schneider and McGrew (2018) for a brief discussion.

5 Carroll used the older Glr and Gsm notation for two factors. These CHC factor labels, where appropriate, have been changed to Gl and Gr (formerly together as Glr), and Gwm as per the most recent iteration of CHC theory (Schneider and McGrew 2018).

6 In Carroll’s (2003) analyses, he used the complete variable names in the correlation matrices presented in that publication (Tables 1.1 and 1.4), but used his own test variable name abbreviations when presenting the results of his factor analysis (Tables 1.2, 1.3, and 1.5). His test name abbreviations are included here for those interested in linking the current discussion with the formal factor analysis output presented in his 2003 chapter. In this section, MC factor loadings from Table 1 are in regular font, while Carroll analysis factor loadings (Table 1.5 in Carroll 2003) are in italic font.

7 As defined by Schneider and McGrew (2018), “*Major* factors are those that represent the core characteristics of the domain, typically represent more complex cognitive processing, tend to display higher loadings on the *g* factor when present in the analysis, and are more predictive and clinically useful. *Minor* factors typically are less useful, less cognitively complex, and less *g*-loaded.” (p. 141; emphasis added).

8 As would be expected, each test’s loadings on the nine CHC factors in the hierarchical *g* and Horn no-*g* models had identical factor loadings (save for a few differences to the second decimal place) and only differed in the specification of the covariance of the first-order latent CHC factors (see Supplementary Materials Section S5, Tables S4 and S6) as correlated factors or as loadings on the second-order *g* factor. Furthermore, inspection of fit statistics found that the MC analysis bifactor (CFI = .890; TLI = .878; RMSEA = .063 [90% CI 0.62–0.65]), hierarchical *g* (CFI = .871; TLI = .862; RMSEA = .067 [90% CI 0.66–0.69]), and Horn no-*g* (CFI = .880; TLI = .868; RMSEA = .066 [90% CI 0.65–0.67]) models did not differ substantially in model fit (see Supplementary Materials Section S4, Tables S3, S5 and S8). Substantive theoretical considerations would be important in determining which of the three models was a “better” model, a discussion beyond the scope of the current paper.

9 Supplementary Materials Section S6 includes a copy of Carroll’s last known recorded thoughts regarding the psychometric *g* factor (copy of hand printed note dated 6-11-03 and a transcribed version). This note includes the referenced personal communication statement.

10 Carroll used the phrase “computer scientists … [as a] shorthand label, as it were, for a wide class of educational research specialists. Not all of those I refer to in this way are necessarily computer scientists in the strict sense”(Carroll 1985, p. 384). Instead, Carroll used this label to characterize a class of researchers as described in this paragraph (i.e., the hard-nosed methodologists).

## Supplementary Materials

The following supporting information can be downloaded at: https://www.mdpi.com/article/10.3390/jintelligence11020032/s1, Section S1 CHC Broad and Narrow Ability Definitions and Codes; Section S2, Descriptions of 46 WJ III Tests; Section S3, McGrew–Carroll Exploratory Factor Analysis (Principal Factoring with Hierarchical Orthogonalization of Factors with Schmid-Leiman Technique; EFA-SL) of 46 WJ III Tests in Ages 14–19 Sample (Completed 05-29-03), Table S1, Best Available Copy of McGrew–Carroll EFA-SL of 46 WJ III Tests (Completed 5-29-03) Using Carroll’s EFA-SL Software and Methods Described in His 1993 Seminal Book, Table S2, Reorganized, Reformatted and Relabeled Version of Table S1; Section S4, Notes on Completion of WJ III 46-test CFA, Table S3, WJ III 46-test CFA Model Fit Statistics (Model Solution in Table 1 in manuscript); Section S5, Supplementary Hierarchical *g* and Horn no-*g* WJ III 46-test CFA, Table S4, WJ III 46-test Hierarchical *g* CFA model, Table S5, WJ III 46-test Hierarchical *g* CFA (Table S4) Model Fit Statistics, Table S6, WJ III 46-test Horn CFA no-*g* Model, Table S7, WJ III 46-test Horn no-*g* Model (Table S6) Latent Factor Correlations, Table S8, WJ III 46-test Horn no-*g* CFA Model (Table S6) Fit Statistics; Section S6, Carroll’s Final Recorded Hand Printed Thoughts Regarding the General Intelligence (*g*) factor; Section S7 CHC Higher-Stratum Design Psychometric Network Analysis Centrality Metrics and Weight Matrices for Reading and Math Models, Table S9, CHC Cognitive–Achievement PNA Centrality Metrics for Reading and Math Models, Table S10, CHC Cognitive–Achievement PNA Weights Matrices for Reading and Math Models; References.
