# Supplementary material for: Carroll’s Three-Stratum (3S) Cognitive Ability Theory at 30 Years: Impact, 3S-CHC Theory Clarification, Structural Replication, and Cognitive–Achievement Psychometric Network Analysis Extension
Source: J Intell. 2023 Feb 6;11(2):32. doi: 10.3390/jintelligence11020032 (PMC9959556; doi:10.3390/jintelligence11020032)
Supplement: Supplementary file 1 [file jintelligence-11-00032-s001.zip › jintelligence-2139899-supplementary.pdf]

Supplementary Materials

Section S1: CHC Broad and Narrow Ability Definitions and Codes

(Abstracted from: Schneider, W. J., and McGrew, K. S. (2012). The Cattell-Horn-Carroll model of intelligence. In D. P. Flanagan and P. L. Harrison (Eds.), *Contemporary intellectual assessment: Theories, tests and issues* (3rd ed., pp. 99–144). Guilford Press.

Narrow abilities with bold font = major ability; regular font = minor ability. If all factor codes are regular font under a broad ability = insufficient data to classify as major or minor (Schneider and McGrew, 2018). *Italic* narrow factor code font designates "tentative" abilities.

Fluid reasoning (*Gf*): The use of deliberate and controlled procedures (often requiring focused attention) to solve novel "on the spot" problems that cannot be solved by using previously learned habits, schemas, and scripts.

- **Induction (I):** The ability to observe a phenomenon and discover the underlying principles or rules that determine its behavior. This ability is also known as rule inference.
- General sequential reasoning (RG): The ability to reason logically using known premises and principles. This ability is also known as deductive reasoning or rule application.
- **Quantitative reasoning (RQ**): The ability to reason with quantities, mathematical relations, and operators.
- *Reasoning Speed (RE):* The ability to reason with quantities, mathematical relations, and operators.
- *Piagetian Reasoning (RP):* Seriation, conservation, classification and other cognitive abilities as defined by Piaget's developmental theory.

Short-term working memory (*Gwm*): The ability to maintain and manipulate information in active attention. The mind's mental "scratchpad" or "workbench."

- **Auditory short-term storage (Wa):** The ability to encode and maintain verbal information in primary memory.
- **Visual-spatial short-term storage (Wv):** The ability to encode and maintain visual information in primary memory.
- **Attentional Control (AC):** The ability to manipulate the spotlight of attention flexibly to focus on task-relevant stimuli and ignore task irrelevant stimuli. Sometimes referred to as spotlight or focal attention, focus, control of attention, executive controlled attention, or executive attention.
- **Working memory capacity (Wc):** The ability to manipulate information in primary memory. Technically not a narrow ability (WMC = short-term storage + AC).

Learning efficiency (*Gl*): The ability the ability to learn, store, and consolidate new information over periods of time measured in minutes, hours, days, and years.

- **Associative memory (MA):** The ability to form a link between two previously unrelated stimuli such that the subsequent presentation of one of the stimuli serves to activate the recall of the other stimuli.
- **Meaningful memory (MM):** The ability to remember narratives and other forms of semantically related information.
- Free recall memory (M6): The ability to recall lists in any order.

Visual-spatial processing (*Gv*): The ability to make use of simulated mental imagery to solve problems. Perceiving, discriminating and manipulating images in the "mind's eye."

- **Visualization (Vz):** The ability to perceive complex visual patterns and mentally simulate how they might look when transformed (e.g., rotated, changed in size, partially obscured, and so forth).
- **Speeded rotation (SR):** The ability to solve problems quickly using mental rotation of simple images. This ability is similar to Vz but is distinct because as it involves the *speed* at which mental rotation tasks can be completed.
- **Imagery (IM):** The ability to voluntarily mentally produce very vivid images of objects, people or events that are not actually present.
- Closure speed (CS): The ability to quickly identify and access a familiar, meaningful visual object stored in long-term memory from incomplete or obscured (e.g., vague, partially obscured, disguised, disconnected) visual cues of the object without knowing in advance what the object is.
- Flexibility of closure (CF): The ability to identify a visual figure or pattern embedded in a complex distracting or disguised visual pattern or array, when one knows in advance what the pattern is.
- Visual memory (MV): The ability to remember complex visual images over short periods of time (less than 30 seconds).
- Spatial scanning (SS): The ability to quickly and accurately survey (visually explore) a wide or complicated spatial field or pattern with multiple obstacles and identify a target configuration or identify a path through the field to a target end point.
- Serial perceptual integration (PI): The ability to recognize an object after only parts of it are shown in rapid succession.
- Length estimation (LE): The ability to visually estimate the length of objects (without using measurement instruments).
- Perceptual illusions (IL): The ability to not be fooled by visual illusions.
- Perceptual alternations (PN): Consistency in the rate of alternating between different visual perceptions.
- Perceptual speed (P): See definition under Gs. P has a secondary loading on *Gv*.

Auditory processing (*Ga*): The ability to discriminate, remember, reason, and work creatively (on) auditory stimuli, which may consist of tones, environmental sounds, and speech units.

- **Phonetic coding (PC):** The ability to distinctly hear phonemes, blend sounds into words, and segment words into parts, sounds, or phonemes.
- Speech sound discrimination (US): The ability to detect and discriminate differences in speech sounds (other than phonemes) under conditions of little or no distraction or distortion.
- Resistance to auditory stimulus distortion (UR): The ability to hear words or extended speech passages correctly under conditions of distortion or background noise.
- **Maintaining and judging rhythm (U8):** The ability to recognize and maintain a musical beat.
- Memory for sound patterns (UM): The ability to retain (on a short-term basis) auditory codes such as tones, tonal patterns, or speech sounds.
- Musical discrimination and judgment (U1 U9): The ability to discriminate and judge tonal patterns in music with respect to melodic, harmonic, and expressive characteristics (phrasing, tempo, harmonic complexity, intensity variations).
- Absolute pitch (UP): The ability to perfectly identify the pitch of tones.
- Sound localization (UL): The ability to localize heard sounds in space.

Comprehension-knowledge (*Gc*): The ability to comprehend and communicate culturally-valued knowledge. *Gc* includes the depth and breadth of both declarative and procedural knowledge and skills such as language, words, and general knowledge developed through experience, learning and acculturation.

- **Language Development (LD):** An intermediate stratum ability to comprehend and communicate using language. The general understanding of spoken language at the level of words, idioms, and sentences.

- **Lexical knowledge (VL):** The knowledge of the definitions of words and the concepts that underlie them. Vocabulary knowledge.
- **General (verbal) information (K0):** The breadth and depth of knowledge that one's culture deems essential, practical, or worthwhile for everyone to know.
- Listening ability (LS): The ability to understand speech. This ability starts with comprehending single words and increases to long complex verbal statements.
- Communication ability (CM): The ability to use speech to communicate effectively.
- Grammatical sensitivity (MY): The awareness of the formal rules of grammar and morphology of words in speech.

__Domain-specific knowledge (*Gkn*)__: The depth, breadth, and mastery of specialized declarative and procedural knowledge (knowledge not all members of a society are expected to have). The *Gkn* domain is likely to contain more narrow abilities than are currently listed in the CHC model.

- **General science information (K1):** The range of scientific knowledge (e.g., biology, physics, engineering, mechanics, electronics).
- **Knowledge of culture (K2):** The range of knowledge about the humanities (e.g., philosophy, religion, history, literature, music, and art).
- Mechanical knowledge (MK): Knowledge about the function, terminology, and operation of ordinary tools, machines, and equipment.
- Foreign language proficiency (KL): Similar to language development (see *Gc*) but in another language.
- Knowledge of signing (KF): The knowledge of finger spelling and signing (e.g., American Sign Language).
- Skill in lip reading (LP): Competence in the ability to understand communication from others by watching the movement of their mouths and expressions.

__Reading and writing (*Grw*):__ The depth and breadth of declarative and procedural knowledge and skills related to written language.

- **Reading comprehension (RC):** The ability to understand written discourse.
- **Reading decoding (RD):** The ability to identify words from text.
- Reading speed (RS): The rate at which a person can read connected discourse with full comprehension. Also listed under *Gs*.
- **Writing ability (WA):** The ability to use written text to communicate ideas clearly.
- Spelling ability (SG): The ability to spell words.
- Writing speed (WS): The ability to copy or generate text quickly. Also listed under Gs and Gps.
- English usage (EU): Knowledge of the mechanics of writing (e.g., capitalization, punctuation, and word usage).

__Quantitative knowledge (*Gq*)__: The depth and breadth of declarative and procedural knowledge related to mathematics. The *Gq* domain is likely to contain more narrow abilities than are currently listed in the CHC model.

- **Mathematical knowledge (KM):** The range of general knowledge, not performance of mathematic operations or the solving of problems.
- **Mathematical achievement (A3):** Measured (tested) mathematics achievement.

__Retrieval fluency (*Gr*):__ The rate and fluency at which individuals can access information stored in long-term memory.

- **Ideational fluency (FI):** The ability to rapidly produce a series of ideas, words, or phrases related to a specific condition or object.
- **Expressional fluency (FE):** The ability to rapidly think of different ways of expressing an idea.

- Associational fluency (FA): The ability to rapidly produce a series of original or useful ideas related to a particular concept.
- Sensitivity to problems/alternative solution fluency (SP): The ability to rapidly think of several alternative solutions to a practical problem.
- Originality/creativity (FO): The ability to rapidly produce original, clever, and insightful responses (expressions, interpretations) to a given topic, situation, or task.
- **Speed of lexical access (LA):** The ability to rapidly retrieve words from an individual's lexicon. Verbal efficiency or automaticity of lexical access. An intermediate stratum level ability.
- **Naming facility (NA):** The ability to rapidly call objects by their names.
- **Word fluency (FW):** The ability to rapidly produce words that share a phonological (e.g., fluency of retrieval of words via a phonological cue) or semantic feature (e.g., fluency of retrieval of words via a meaning-based representation).
- Figural fluency (FF): The ability to rapidly draw or sketch as many things (or elaborations) as possible when presented with a nonmeaningful visual stimulus (e.g., a set of unique visual elements).
- Figural flexibility (FX): The ability to rapidly draw different solutions to figural problems.

Processing speed (*Gs*): The ability to control attention to automatically, quickly and fluently perform relatively simple repetitive cognitive tasks. Attentional fluency or attentional speediness

- **Perceptual speed (P):** An intermediate stratum level ability that can be defined as the speed and fluency with which similarities or differences in visual stimuli (e.g., letters, numbers, patterns, etc.) can be searched and compared in an extended visual field.
- **Perceptual speed-search (Ps):** The speed and fluency of searching or scanning an extended visual field to locate one or more simple visual patterns
- **Perceptual speed-compare (Pc):** The speed and fluency of looking up and comparing visual stimuli that are side-by-side or more widely separated in an extended visual field.
- **Number facility (N):** The speed, fluency and accuracy in manipulating numbers, comparing number patterns, or completing basic arithmetic.
- Reading speed (fluency) (RS): The speed and fluency of reading text with full comprehension. Also listed under *Grw*.
- Writing speed (fluency) (WS): The speed and fluency of generating or copying words or sentences. Also listed under *Grw* and *Gps*.

Reaction and decision speed (*Gt*): The speed of making very simple decisions or judgments when items are presented one at a time.

- Simple reaction time (R1): Reaction time to the onset of a single visual or auditory stimulus.
- Choice reaction time (R2): Reaction time when a very simple choice must be made.
- Inspection time (IT): The speed at which differences in visual stimuli can be perceived.
- Semantic processing speed (R4): Reaction time when a decision requires some very simple encoding and mental manipulation of the stimulus content.
- Mental comparison speed (R7): The reaction time required when stimuli must be compared for a particular characteristic or attribute.

Psychomotor speed (*Gps*): The ability to perform skilled physical body motor movements (e.g., movement of fingers, hands, legs) with precision, coordination, fluidity or strength.

- Speed of limb movement (R3): The speed of arm and leg movement. This speed is measured after the movement is initiated. Accuracy is not important.

- Writing peed (fluency) (WS): The speed at which written words can be copied. Also listed under *Grw* and *Gps*.
- Speed of articulation (PT): The ability to rapidly perform successive articulations with the speech musculature.
- Movement time (MT): The time taken to physically move a body part (e.g., a finger) to make the required response, after a decision or choice has been made, in an elementary cognitive task.

Psychomotor abilities (*Gp*): The ability to perform skilled physical body motor movements (e.g., movement of fingers, hands, legs) with precision, coordination, or strength. The *Gp* domain is likely to contain more narrow abilities than are currently listed in the CHC model.

- Manual dexterity (P1): The ability to make precisely coordinated movements of a hand or a hand and the attached arm.
- Finger dexterity (P2): The ability to make precisely coordinated movements of the fingers (with or without the manipulation of objects).
- Static strength (P3): The ability to exert muscular force to move (push, lift, pull) a relatively heavy or immobile object.
- Gross body equilibrium (P4): The ability to maintain the body in an upright position in space or regain balance after balance has been disturbed.
- Multilimb coordination (P6): The ability to make quick specific or discrete motor movements of the arms or legs.
- Arm-hand steadiness (P7): The ability to precisely and skillfully coordinate arm–hand positioning in space.
- Control precision (P8): The ability to exert precise control over muscle movements, typically in response to environmental feedback (e.g., changes in speed or position of object being manipulated).
- Aiming (AI): The ability to precisely and fluently execute a sequence of eye–hand coordination movements for positioning purposes.

Olfactory abilities (*Go*): The ability to detect and process meaningful information in odors. The *Go* domain is likely to contain more narrow abilities than are currently listed in the CHC model.

- Olfactory memory (OM): The ability to recognize previously encountered distinctive odors.

Tactile (haptic) abilities (*Gh*): The ability to detect and process meaningful information in haptic (touch) sensations. It includes perceiving, discriminating and manipulating touch stimuli. Currently there are no well-supported narrow *Gh* cognitive ability factors.

Kinesthetic abilities (*Gk*): The ability to detect and process meaningful information in proprioceptive sensations. Currently there are no well-supported narrow *Gk* cognitive ability factors within *Gk.*

Emotional intelligence (*Gei*): The ability to perceive emotions expressions, understand emotional behavior, and solve problems using emotions.

- ***Emotion perception (Ep):*** The ability to accurately recognize emotions in the face, voice, and behavior.
- ***Emotion knowledge (Ek):*** Knowledge of the antecedents of emotions and the consequences of emotional expression.
- *Emotion management (Em):* The ability to regulate one's emotions deliberately and adaptively.
- *Emotion utilization (Eu)*: The ability to make adaptive use of emotion, especially to facilitate reasoning.

Section S2: Descriptions of 46 WJ III Tests

Fluid reasoning (Gf) tests

Concept Formation: Measures the ability to identify and state the rule for a concept about a set of colored geometric figures when shown instances and non-instances of the concept. This is a "learning" test with corrective feedback and reinforcement of correct answers provided to the subject.

Analysis-Synthesis: Measures the ability to analyze the components of an incomplete logic puzzle and to determine and name the missing components. This is a "learning" test with corrective feedback and reinforcement of correct answers provided to the subject. The test represents an underlying math logic system.

Number Series: Measures the ability to identify the quantitative principle that underlies a sequence of numbers in a series of numbers and then apply the principle to supply a missing number in the series.

Number Matrices: Measures the ability to identify the quantitative principle that underlies a series of numbers in matrices and then apply the principle to supply a missing number in the matrix.

Verbal Knowledge-Comprehension (Gc) tests

Verbal Comprehension: Measures knowledge of word meanings. The test consists of four subtests. *Picture Vocabulary* - the subject must name familiar and unfamiliar pictured objects. *Oral Vocabulary: Synonyms* - the subject must say a word similar in meaning to the word presented. *Oral Vocabulary: Antonyms* - the subject must say a word that is opposite in meaning to the word presented. *Verbal Analogies* - the subject must complete phrases with words that indicate appropriate analogies.

General Information: Measures knowledge of the common or typical characteristics of certain objects. The test has two component subtests: "*what*" and "*where*" questions. The questions are presented orally, and the subject must state the answer to "where you would find...." and "what you would do with..." questions.

Oral Comprehension: Measures the ability to listen to a short tape/CD-recorded passage and to verbally supply the single word missing at the end of the passage.

Academic Knowledge: Comprised of three subtests (*Science, Social Studies, Humanities*) that measures the subject's knowledge in various areas of the biological and physical sciences, history, geography, government, economics, art, music, and literature.

Picture Vocabulary: Measures knowledge of word meanings. The subject must name familiar and unfamiliar pictured objects.

Story Recall: Measures the ability to recall increasingly complex stories presented orally to the subject. The subject is asked to tell back as much of the story as they can. The score is based on the number of correctly recalled story elements.

Story Recall-Delayed Recall: Measures the ability to recall (after 1 to 8 days) the stories presented in Story Recall.

Visual-spatial (Gv) tests

Spatial Relations: Measures the ability to visually match and combine shapes. The subject must select from a series of shapes, the component parts composing a given whole shape.

Block Rotation: Measures the ability to recognize two geometric 3-D designs, in a row of five, that are identical, although rotated to a different visual perspective from the target geometric 3-D design. Below the target stimulus are five pictures of geometric shapes or three-dimensional block strings that have been rotated in space. The subject must identify which two drawings are replications of the target item.

Picture Recognition: Measures the ability to recognize a subset of previously presented pictures within a larger set of pictures.

Visual Closure: Measures the ability to name a drawing or picture of a simple object that is represented by disconnected lines. The test requires the subject to visually combine the disconnected lines into a meaningful whole.

Planning: Measures the ability to plan a tracing route that covers as many segments of a dotted line drawing as possible without lifting the pencil or tracing over the same segment twice. The test requires "forward thinking" in that the subject is required to plan a sequence of steps prior to initializing the plan.

Auditory processing (Ga) tests

Sound Blending: Measures the ability to perform auditory synthesis on segments of speech. After hearing the recorded parts (syllables and/or phonemes) of a word from a tape/CD, the subject must "blend" the parts together to make a whole word (synthesis task).

Incomplete Words: Measures the ability to perform auditory closure on segments of speech. After hearing a recorded word from tape/CD with one or more phonemes missing, the subject must name the complete word (analysis task).

Sound Patterns-Voice: Measures the ability to indicate whether pairs of complex sounds presented with a tape/CD are the same or different. The sound patterns resemble human speech sounds. The pairs may differ in pitch, rhythm, or sound content.

Auditory Attention: Measures the ability to discriminate similar sounding words. An audio tape/CD is used to present the words to the subject with increasing levels of background noise as a distracter.

Sound Awareness: Measures oral sound analysis skills at the preschool and primary level of development. It consists of four subtests - *Rhyming, Substitution, Deletion, and Reversal*. The items are presented orally by the examiner, or by tape/CD, and the subject responds orally.

Sound Patterns-Music: Measures the ability to indicate whether pairs of musical sounds are the same or different. The pairs may differ in pitch, rhythm, or sound content. The sounds are presented with a tape/CD.

Long-term storage and retrieval (Glr) tests

Visual-Auditory Learning: Measures the ability to associate new visual symbols (rebuses) with familiar words in oral language and to translate a series of symbols presented as a reading passage (a visual-auditory association task). This is a "learning" test where corrective feedback is provided to the subject.

Memory for Names: Measures the ability to learn associations between unfamiliar auditory and visual stimuli (an auditory—visual association task). The task requires learning the names of a series of space creatures.

Visual-Auditory Learning-Delayed Recall: Measures the ability to recall and relearn (after 1 to 8 days) the symbols (rebuses) presented in Visual-Auditory Learning. This is a "relearning" task as the subject relearns forgotten associations. Corrective feedback is provided to the subject during the task.

Memory for Names-Delayed Recall: Measures the ability to recall (after 1 to 8 days) the space creatures presented in Memory for Names.

Retrieval Fluency: Measures fluency in retrieving the names of objects. The subject is asked to state as many items as they can of three different types, "things to eat or drink", "names of people", and "animals.

Working memory (Gwm) tests

Memory for Sentences: Measures the ability to remember and repeat simple words, phrases, and sentences presented by a tape/CD player.

Memory for Words: Measures the ability to repeat lists of unrelated words in the correct sequence; words are presented by audio tape/CD.

Numbers Reversed: Measures the ability to repeat a series of random numbers backward. The number sequences are presented by audio tape/CD.

Auditory Working Memory: Measures the ability to retain two types of orally presented information and then repeat them in a specified order. The subject is presented a mixed series of words and digits and is asked to rearrange them by first saying the words in order and then the numbers. The task requires divided attention as the subject must perform two different mental operations simultaneously.

Understanding Directions: Measures comprehension of linguistic concepts (receptive language). The subject is asked to follow increasing complex oral directions by pointing to different items in a picture.

Processing speed (Gs) tests

Visual Matching: Measures the ability to quickly locate and circle the two identical numbers in a row of six numbers. The task proceeds in difficulty from single-digit numbers to triple-digit numbers and has a 3-minute time limit.

Cross Out: Measures the ability to quickly scan and compare visual information. The subject must mark the five drawings in a row of 20 drawings that are identical to the first drawing in the row. The subject is given a 3-minute time limit to complete as many rows of items as possible.

Decision Speed: Measures the ability to rapidly scan a row of pictures and decide which of the two drawings are the most related. The decisions become slightly more abstract as the test progresses. The subject is instructed to complete as many rows of drawings as possible within a three-minute time limit.

Rapid Picture Naming: Measures the ability to rapidly identify and orally name pictures of common objects. The stimulus pictures are presented in rows of five. The test has a two-minute time limit.

Pair Cancellation: Measures the capacity for sustained attention (vigilance). The subject is presented with rows that contain repeating pictures of a dog and a ball (in no particular sequence) and must circle all instances of when the "ball is followed by the dog". The test has a three-minute time limit.

Mathematics (Gq) tests

Calculation: Measures the ability to perform mathematical calculations ranging from simple addition to calculus. The subject is not required to make any decisions about what operations to use or what data to include.

Applied Problems: Measures the ability to analyze and solve problems in mathematics. The subject must decide not only the appropriate mathematical operations to use but also which of the data to include in the calculation.

Quantitative Concepts: Measures the subject's mathematical vocabulary, concepts, and quantitative reasoning. The test consists of a mixture of number series items and items requiring the subject to display mathematical knowledge. The number series items require the subject to identify the underlying numerical relation in a series of numbers and then apply this principle by supplying a missing number in the series.

Math Fluency: Measures the ability to quickly perform single-digit addition, subtraction, and multiplication facts. The subject is presented a series of simple arithmetic problems on a worksheet. The subject has two minutes to complete as many problems as possible.

Reading (Grw-R) tests

Letter-Word Identification: Measures the subject's reading skills in identifying isolated letters and words. It is not necessary that the subject knows the meaning of any words correctly identified.

Word Attack: Measures the ability to apply phonic and structural analysis skills to the pronunciation of unfamiliar printed words. The subject reads aloud letter combinations that are linguistically logical in English but that do not form actual words (nonsense word), or words that constitute low-frequency words in the English language.

Passage Comprehension: Measures the subject's skill in reading a short passage and identifying a missing key word. In this modified cloze procedure, the subject must exercise a variety of comprehension and vocabulary skills.

Reading Vocabulary: Measures subject's skill in reading and understanding the meanings of words. Has three subtests. *Synonyms* - the subject must read a word and provide a word similar in meaning to the word presented. *Antonyms* - the subject must read a word and provide a word that is opposite in meaning to the word presented. *Analogies* - the subject must read an analogy and provide the missing word.

Reading Fluency: Measures the ability to quickly comprehend the correctness of simple sentences. The subject is presented a series of simple sentences and must circle whether each sentence is true or false. The subject is required to complete as many items as possible within a 3-minute time limit.

Writing (Grw-W) tests

Spelling: Measures the ability to write correct spellings of orally presented words.

Editing: Measures the ability to identify, and indicate how to correct, mistakes in typewritten passages. The error in the passage may be incorrect punctuation or capitalization, inappropriate word usage, or a misspelling.

Writing Samples: Measures the ability to write responses to a variety of demands. The subject must phrase and present written sentences that are evaluated with respect to the quality of expression. The subject is not penalized for errors in the basic mechanics of writing (spelling; punctuation).

Writing Fluency: Measures the ability to formulate and write simple sentences quickly. This subtest has a 7-minute time limit.

Spelling of Sounds: Measures the ability to listen to a nonsense word and produce a written response representing the likely spelling of that word if it were a real English word. It is a measure of the subject's comprehension of the "alphabetic principle." The subject is presented the nonsense word from an audio tape/CD and is asked to write it.

Section S3: McGrew-Carroll Exploratory Factor Analysis (Principal Factoring with Hierarchical Orthogonalization of Factors with Schmid-Leiman Technique; EFA-SL) of 46 WJ III Tests in Ages 14-19 Sample (Completed 05-29-03)

Table S1

Best Available Copy of McGrew-Carroll EFA-SL of 46 WJ III Tests (Completed 05-29-03) Using Carroll's EFA-SL Software and Methods Described in His 1993 Seminal Book

W00031

5-29-03 WJ3+DS Carroll EFA-SL analysis of ages 14-19 norm data-by Kevin McGrew, IAP – final solution 7-5-03 – Factor labels revised

1

**REORDERED FACTOR MATRIX \WORK\WJ3046_H.TB

WJ3 DS WOODCOOCK ET AL (2001 2003)
CORR MATRIX BASED ON AGE 14 TO 19
COG AND ACH 47 VARIABLES
N= 1618

	V#		1	2	3	4	5	6
		**FACTOR 1 : O2;F1 2G-GENERAL INTELLIGENCE......; ORDER 2**						
		**FACTOR 2 : O1;F3 GRW-RD/SA/EU-PHONEME-GRAPHEME KNOWLEDGE?; ORDER 1**						
1 +	31	WORD ATTACK..................	.649	**.484**	-.006	-.015	-.029	.019
2 +	37	SPELLING OF SOUNDS...........	.697	**.397**	.013	.015	-.016	-.014
3 +	20	LETTER WORD ID...............	.733	**.352**	.218	.005	.010	-.010
4 +	26	SPELLING.....................	.665	**.307**	.100	.046	.021	.001
5 +	30	WRITING SAMPLES..............	.624	.135	.160	.063	.003	.068
		**FACTOR 3 : O1;F1 GC(1C)-CRYSTALLIZED KNOWLEDGE; ORDER 1**						
6 +	10	GENERAL INFORMATION..........	.697	.005	**.585**	-.056	.004	-.015
7 +	36	ACADEMIC KNOWLEDGE...........	.707	-.010	**.577**	-.000	-.012	.001
8 +	1	VERBAL COMPREHENSION.........	.741	.004	**.556**	.018	-.004	.007
9 +	32	PICTURE VOCABULARY...........	.555	-.061	**.553**	-.000	-.018	-.073
10 +	35	READING VOCABULARY...........	.675	.132	**.495**	-.022	-.004	.021
11 +	29	APPLIED PROBLEMS.............	.680	-.007	**.409**	-.037	-.034	.115
12 +	22	STORY RECALL.................	.708	-.044	**.379**	.091	-.003	.177
13 +	33	ORAL COMPREHENSION...........	.569	-.045	**.363**	-.003	.019	.072
14 +	28	PASSAGE COMPREHENSION........	.617	.088	**.300**	-.014	.043	.001
15 +	34	EDITING......................	.632	***.228***	***.276***	.008	.032	-.024
16 +	38	SOUND AWARENESS..............	.746	.123	***.260***	.074	-.014	.172
17 +	14	ANALYSIS-SYNTHESIS...........	.612	-.015	***.226***	.092	.074	.133
		**FACTOR 4 : O1;F8 MA[GLR]-ASSOCIATIVE MEMORY (SOME MV?[GLR]-VIS MEM?)..; ORDER 1**						
18 +	39	MEMORY FOR NAMES.............	.462	-.010	-.022	**.497**	-.003	.003
19 +	2	VISUAL AUDITORY LEARNING......	.591	.020	.014	**.467**	-.019	.061
20 +	12	PICTURE RECOGNITION..........	.353	-.037	.013	***.284***	.141	.008
21 +	41	SOUND PATTERNS-VOICE.........	.433	.097	-.069	***.235***	-.039	-.008
22 +	40	VISUAL CLOSURE...............	.221	.012	.004	***.197***	.173	-.006
		**FACTOR 5 : O1;F2 GS(1S)-PROCESSING SPEED .... ORDER 1**						
23 +	15	DECISION SPEED...............	.416	-.011	-.002	-.021	**.714**	.105
24 +	19	PAIR CANCELLATION............	.482	.008	-.068	-.014	**.689**	.014
25 +	6	VISUAL MATCHING..............	.539	.076	-.054	.009	**.550**	-.074
26 +	44	CROSS OUT....................	.526	.008	-.001	.029	**.544**	-.026
27 +	17	RAPID PICTURE NAMING.........	.388	-.096	.007	.146	**.315**	-.125
28 +	11	RETRIEVAL FLUENCY............	.470	-.075	***.206***	.028	***.221***	-.009
29 +	27	WRITING FLUENCY..............	.574	.049	.092	-.004	***.210***	.023
		**FACTOR 6 : O1;F9 GF?(1F?)-FLUID REASONING?....; ORDER 1**						
30 +	5	CONCEPT FORMATION............	.674	.008	.011	.199	.021	**.499**
31 +	23	UNDERSTANDING DIRECTIONS......	.778	.003	.174	.023	**.302**	**.492**
		FACTOR 7 : O1;F10 RE?[GF]-SPEED OF REASONING?....; ORDER 1						
32 +	25	MATH FLUENCY.................	.565	.006	.008	-.006	*.251*	.091
33 +	21	READING FLUENCY..............	.662	-.001	.080	-.004	*.267*	.008
		FACTOR 8 : O1;F6 GSM(1X)-SHORT-TERM MEMORY....; ORDER 1						
34 +	45	MEMORY FOR SENTENCES.........	.610	.015	***.280***	-.004	-.019	.011
35 +	16	MEMORY FOR WORDS.............	.557	.110	.083	.016	-.010	.017
36 +	7	NUMBERS REVERSED.............	.577	.144	.004	.063	.039	.011
37 +	9	AUDITORY WORKING MEMORY.......	.595	.060	.160	-.016	.089	-.015
		FACTOR 9 : O1;F4 RQ[GF]+GQ-A3 QUANTITATIVE REASONING+QUANT KNOWLEDGE ORDER 1						
38 +	42	NUMBER SERIES................	.658	-.014	**.350**	.013	.022	-.024
39 +	43	NUMER MATRICES...............	.635	.030	**.313**	.012	-.001	.014
40 +	24	CALCULATION..................	.556	.015	.271	.001	.004	.045
		FACTOR 10 : O1;F5 GV(1V)-VISUAL-SPATIAL PROC..; ORDER 1						
41 +	3	SPATIAL RELATIONS............	.434	.018	.010	.165	.103	.018
42 +	18	PLANNING.....................	.296	-.007	-.015	.108	.015	.059
43 +	46	BLOCK ROTATION...............	.362	-.015	.054	***.195***	.026	-.024
		FACTOR 11 : O1;F7 GA(1U)-AUDITORY PROCESSING..; ORDER 1						
44 +	4	SOUND BLENDING...............	.519	.141	.002	.193	.026	.007
45 +	13	AUDITORY ATTENTION...........	.369	-.004	-.002	.116	***.259***	.025
46 +	8	INCOMPLETE WORDS.............	.410	.059	.087	.104	.026	-.046
		SMSQ	15.482	.825	2.862	.880	2.155	.657

5-29-03 WJ3+DS Carroll EFA-SL analysis of ages 14-19 norm data-by
Kevin McGrew, IAP – final solution 7-5-03 – Factor labels revised

v#		Test	7	8	9	10	11	H^2
		FACTOR 1 : O2;F1 2G-GENERAL INTELLIGENCE......; ORDER 2						
		FACTOR 2 : O1;F3 GRW-RD/SA/EU-PHONEME-GRAPHEME KNOWLEDGE?; ORDER 1						
1 +	31	WORD ATTACK..................	-.014	-.031	-.117	-.004	.110	.684
2 +	37	SPELLING OF SOUNDS...........	.037	.015	.014	-.012	**.395**	.802
3 +	20	LETTER WORD ID...............	-.032	.031	-.133	-.114	-.030	.743
4 +	26	SPELLING.....................	.132	-.083	.009	-.133	-.018	.591
5 +	30	WRITING SAMPLES..............	.104	.028	-.009	.025	.044	.456
		FACTOR 3 : O1;F1 GC(1C)-CRYSTALLIZED KNOWLEDGE; ORDER 1						
6 +	10	GENERAL INFORMATION..........	.026	-.018	.003	-.042	.030	.835
7 +	36	ACADEMIC KNOWLEDGE...........	-.024	-.006	.088	-.093	-.034	.850
8 +	1	VERBAL COMPREHENSION.........	-.013	.004	-.013	-.000	.032	.860
9 +	32	PICTURE VOCABULARY...........	-.005	-.027	-.001	.017	.129	.641
10 +	35	READING VOCABULARY...........	-.161	.098	-.031	-.281	-.075	.839
11 +	29	APPLIED PROBLEMS.............	.019	-.051	**.410**	-.004	-.047	.818
12 +	22	STORY RECALL.................	.060	.026	.026	-.000	-.030	.693
13 +	33	ORAL COMPREHENSION...........	.072	.107	.000	.003	-.011	.480
14 +	28	PASSAGE COMPREHENSION........	.021	.078	.025	.016	.021	.488
15 +	34	EDITING......................	.031	-.017	-.001	-.281	-.125	.625
16 +	38	SOUND AWARENESS..............	-.032	-.012	.124	-.011	.061	.695
17 +	14	ANALYSIS-SYNTHESIS...........	-.071	.060	***.185***	.111	-.094	.521
		FACTOR 4 : O1;F8 MA[GLR]-ASSOCIATIVE MEMORY (SOME MV?[GLR]-VIS MEM?)..; ORDER 1						
18 +	39	MEMORY FOR NAMES.............	.008	-.025	.002	-.038	.001	.463
19 +	2	VISUAL AUDITORY LEARNING......	.005	.002	.023	.015	.002	.572
20 +	12	PICTURE RECOGNITION..........	-.004	.045	-.117	.029	-.093	.252
21 +	41	SOUND PATTERNS-VOICE.........	.091	-.014	.066	***.205***	***.186***	.348
22 +	40	VISUAL CLOSURE...............	-.122	-.056	-.123	.023	.034	.152
		FACTOR 5 : O1;F2 GS(1S)-PROCESSING SPEED .... ORDER						
23 +	15	DECISION SPEED...............	-.165	-.006	-.160	-.109	.127	.775
24 +	19	PAIR CANCELLATION............	.028	-.032	.006	.015	.034	.715
25 +	6	VISUAL MATCHING..............	.079	.041	.095	-.018	-.039	.626
26 +	44	CROSS OUT....................	-.012	.111	-.014	.083	-.039	.595
27 +	17	RAPID PICTURE NAMING.........	***.260***	.102	-.018	-.026	.098	.385
28 +	11	RETRIEVAL FLUENCY............	.152	.110	.022	-.145	.009	.375
29 +	27	WRITING FLUENCY..............	***.236***	.068	-.007	.025	.087	.453
		FACTOR 6 : O1;F9 GF?(1F?)-FLUID REASONING?....; ORDER 1						
30 +	5	CONCEPT FORMATION............	.015	-.001	-.036	.188	-.030	.782
31 +	23	UNDERSTANDING DIRECTIONS......	-.155	.001	.026	.002	.027	.995
		--						
		**FACTOR 7 : O1;F10 GR(1R) – BROAD RETRIEVAL FLUENCY? ORDER 1**						
32 +	25	MATH FLUENCY.................	**.464**	-.033	**.361**	.001	.003	.738
33 +	21	READING FLUENCY..............	**.452**	.030	.008	.128	.002	.739
		**FACTOR 8 : O1;F6 GSM(1X)-SHORT-TERM MEMORY....; ORDER 1**						
34 +	45	MEMORY FOR SENTENCES.........	.019	**.441**	-.008	-.035	-.000	.648
35 +	16	MEMORY FOR WORDS.............	-.006	**.435**	.028	.029	.086	.528
36 +	7	NUMBERS REVERSED.............	.006	**.323**	.166	-.004	.041	.493
37 +	9	AUDITORY WORKING MEMORY.......	.002	***.281***	***.199***	.003	.172	.539
		**FACTOR 9 : O1;F4 RQ[GF]+GQ-A3 QUANTITATIVE REASONING+QUANT KNOWLEDGE ORDER 1**						
38 +	42	NUMBER SERIES................	.013	.020	**.454**	.036	-.003	.765
39 +	43	NUMER MATRICES...............	-.043	.030	**.411**	.017	.024	.675
40 +	24	CALCULATION..................	.074	-.081	**.397**	-.027	-.086	.563
		**FACTOR 10 : O1;F5 GV(1V)-VISUAL-SPATIAL PROC..; ORDER 1**						
41 +	3	SPATIAL RELATIONS............	.004	-.027	.004	**.425**	-.014	.408
42 +	18	PLANNING.....................	.099	-.112	.117	**.321**	.045	.244
43 +	46	BLOCK ROTATION...............	-.066	.021	.107	**.304**	-.032	.283
		**FACTOR 11 : O1;F7 GA(1U)-AUDITORY PROCESSING..; ORDER 1**						
44 +	4	SOUND BLENDING...............	.013	.012	-.018	.005	**.540**	.620
45 +	13	AUDITORY ATTENTION...........	-.035	.013	.009	-.029	**.439**	.413
46 +	8	INCOMPLETE WORDS.............	.071	.071	-.051	.041	**.297**	.295
SMSQ			.752	.689	1.091	.734	.935	27.061

Handwritten notes: beside WRITING SAMPLES (.456): "no salient factor loadings – JC Ho – idea production (Gr)?"; beside UNDERSTANDING DIRECTIONS (.995, circled): "highest H^2"; beside FACTOR 7 heading: "– Gs-Ach?"

5-29-03 WJ3+DS Carroll EFA-SL analysis of ages 14-19 norm data-by Kevin McGrew, IAP – final solution 7-5-03 – Factor labels revised

**** ORTHOGONALIZED MATRIX AT ORDER 2:**

V#		G	H^2
3-GRW	PHONEME-GRAPHEME KNOWLEDGE	.838	.702
1-GC(1C)	CRYSTALLIZED KNOWLEDGE	.792	.627
8-MA(GLR)	ASSOCIATIVE MEMORY	.726	.528
2-GS(1S)	PROCESSING SPEED	.624	.390
9-GF(1F)?	FLUID REASONING?	.595	.354
10-GR(1R)	BROAD RETRIEVAL FLUENCY	.532	.284
6-GSM	SHORT-TERM MEMORY	.473	.224
4-RQ(GF)+GQ-A3	QUANTITATIVE REASONING+KNOWLEDGE	.372	.138
5-GV(1V)	VISUAL-SPATIAL PROCESSING	.303	.092
7-GA	AUDITORY PROCESSING	.112	.013
	SMSQ	3.351	3.351

The McGrew-Carroll EFA-SL analyses (Table S1) was based on the published correlation matrix of tests for the age 14-19 WJ III norm sample (n = 1616). The analysis was completed as per Carroll's description of the methods and latest version of the software he used in his 1993 book (see chapter 3). The original pool of 51 WJ III tests were selected for the EFA-SL analysis. The completion of the first run of the data (05-28-03) produced a singular matrix. This was determined to be due to high communalities (≥ .90) for five tests (Story Recall-Delayed Recall, Visual-Auditory Learning-Delayed Recall, Memory for Names-Delayed Recall, Quantitative Concepts, Sound Patterns-Music). These five tests were removed from the analysis.

Tests with substantial factor loadings, as defined by Carroll (1993, 2003), are designated by bold font (≥ .30) or italic bold font (≤ .30) in the EFA-SL output (Table S1). The factor label names reported in the original output (Table S1) reflect the preliminary or "working" factor names assigned to each factor—not necessarily the final factor names that would have been used in a planned coauthored journal manuscript. For example, factor 7 was tentatively labeled "broad retrieval fluency?" which would correspond to Gr. However, the WJ III Retrieval Fluency test, a classic Gr measure (Carroll, 1993; Schneider and McGrew, 2018), did not display a salient factor loading (.152) on this factor, while the highest two loading tests were the academic fluency tests of Math Fluency (.464) and Reading Fluency (.452). Thus, it was noted that this factor may represent and academic speed or fluency factor.

A reorganized, reformatted, and relabeled version of Table S1 is presented in Table S2. The final factor names in Table S2 reflect the most likely CHC factor names as per McGrew and Schneider (2018). The organization of Table S2 reflects an adaptation of Carroll's standard factor analysis output (e.g., see Table 3.3, p. 104-106 in Carroll, 1993) as presented in Table S1. This modified organization is like a table originally presented by McGrew (1997) which was approved by Carroll. Only salient loadings (bold and bold italic font) from the original output (Table S1) are included (see Carroll, 1993, Chapter 3 for discussion).

Table S2

Reorganized, Reformatted and Relabeled Version Table S1

	First-order factors												
	1A	1B	1C	1D	1E	1F	1G	1H	1I	1J	1K	*2nd-order*	
	g	Grw	Gc	Gl	Gsc	Gf	Gsa	Gwm	Gq	Gv	Ga	*g factor*	h^2
**1B: Grw-Phoneme-grapheme knowledge**												**0.84**	**0.70**
Word Attack	0.65	0.48											0.68
Spelling of Sounds	0.70	0.40									0.40		0.80
Letter-Word ID	0.73	0.35											0.74
Spelling	0.66	0.31											0.59
Writing Samples	0.62												0.46
**1C: Gc-Crystallized Intelligence**												**0.79**	**0.63**
General Information	0.70		0.59										0.84
Academic Knowledge	0.71		0.58										0.85
Verbal Comprehension	0.74		0.56										0.86
Picture Vocabulary	0.56		0.55										0.64
Reading Vocabulary	0.68		0.50										0.84
Applied Problems	0.68		0.41						0.41				0.82
Story Recall	0.71		0.38										0.69
Oral Comprehension	0.57		0.36										0.48
Passage Comprehension	0.62		0.30										0.49
Editing	0.63	0.23	0.28										0.63
Sound Awareness	0.75		0.26										0.70
Analysis-Synthesis	0.61		0.23						0.19				0.52
**1D: Gl-Associative Memory**												**0.73**	**0.53**
Memory for Names	0.46			0.50									0.46
Visual Auditory Learning	0.59			0.47									0.57
Picture Recognition	0.35			0.28									0.25
Sound Patterns Voice	0.43			0.24						0.21	0.19		0.35
Visual Closure	0.22			0.20									0.15
**1E: Gsc-Processing Speed: Cognitive**												**0.62**	**0.39**
Decision Speed	0.42				0.71								0.78
Pair Cancellation	0.48				0.69								0.72
Visual Matching	0.54				0.55								0.63
Cross Out	0.53				0.54								0.60
Rapid Picture Naming	0.39				0.32		0.26						0.39
Retrieval Fluency	0.47		0.21		0.22								0.38
Writing Fluency	0.57				0.21		0.24						0.45
**1F: Gf-Fluid Reasoning**												**0.60**	**0.35**
Concept Formation	0.67					0.50							0.78
Understanding Directions	0.78				0.30	0.49							1.00
**1G: Gsa-Processing Speed: Achievement**												**0.53**	**0.28**
Math Fluency	0.56				0.25		0.46		0.36				0.74
Reading Fluency	0.66				0.27		0.45						0.74
**1H: Gwm-Working Memory Capacity**												**0.47**	**0.22**
Memory for Sentences	0.61		0.28					0.44					0.65
Memory for Words	0.56							0.44					0.53
Numbers Reversed	0.58							0.32					0.49
Auditory Working Memory	0.60							0.28	0.20				0.54
**1I: Gq-Quantitative Knowledge/Reasoning**												**0.37**	**0.14**
Number Series	0.66		0.35						0.45				0.77
Number Matrices	0.64		0.31						0.41				0.68
Calculation	0.56								0.40				0.56
**1J: Gv-Visual-Spatial Processing**												**0.30**	**0.09**
Spatial Relations	0.43									0.43			0.41
Planning	0.30									0.32			0.24
Block Rotation	0.36									0.30			0.28
**1K: Ga-Auditory Processing**												**0.11**	**0.01**
Sound Blending	0.52										0.54		0.62
Auditory Attention	0.37				0.26						0.44		0.41
Incomplete Words	0.41										0.30		0.30
**Sum of Squares:**	15.48	0.83	2.86	0.88	2.16	0.66	0.75	0.69	1.09	0.73	0.94		27.06

Section S4: Notes on Completion of WJ III 46-test CFA

The CFA analyses completed in the summer of 2022 was based on the same WJ III norm sample age group but included 23 additional subjects (n = 1641). The reason for the minor sample size difference is due to the original WJ III technical manual correlation matrices being based on *preliminary* "research standard scores derived from a simple *z*-score transformation of W scores at each of the 26 age groups" (McGrew and Woodcock, 2001, p. 60). The CFA analyses reported here used the *final* norm data file that included the publication standard scores generated with more sophisticated and precise procedures as described in the WJ III technical manual (McGrew and Woodcock, 2001, p. 60 and Chapter 2). The difference of 23 subjects is likely due to: (1) the preliminary (cruder) standard score generation procedure excluding some subjects, (2) the final data files with the norm-based standard scores including a few more subjects (due to final data file cleaning), or (3) some combination of both. It is assumed that the increase of 23 subjects in the final publication standard score analysis did not appreciably change the underlying relations between the 46 tests used in both the EFA-SL and CFA.

All test variables with salient loadings from the McGrew-Carroll EFA-SL (Table S1 and S2) were specified on the appropriate factors for the first model run. Since the Writing Samples test displayed no salient factor loading in the EFA-SL analysis, attempts were made to specify it to load on the Grw, Gc, and Gr factors (Carroll hypothesized that the generation of written text may require idea production) in isolation, or in various combinations. Writing Samples only demonstrated a salient loading on Grw and was so specified in the subsequent model runs. Several tests either displayed non-significant or negative loadings during the first model run. These tests were removed from the respective factors in the second model run—Gc (Applied Problems, Analysis-Synthesis, Editing, Retrieval Fluency, Number Series, Number Matrices, Sound Awareness); Gl (Sound Patterns-Voice, Block Rotation). The Sound Awareness test did not display any significant positive loading on either the Ga, Grw, or Ga factors (all attempts to specify Sound Awareness on these factors either produced non-significant or negative parameter values). Sound Awareness was removed from the CFA analyses. After each analysis, the modification indices were inspected for possible additional test cross-loadings. The addition of factor cross-loadings were made conservatively and added only if they made substantive sense. Only two cross-loading modification specifications were made—Spelling of Sounds on Ga and Math Fluency on Gq. The model fit statistics for the CFA model presented in the main body of this paper (Table 1) are presented below.

Table S3

WJ III 46-test CFA Model Fit Statistics (Model Solution in Table 1 in manuscript)

Chi-square test

Model	X²	df	p
Baseline model	56783.021	1035	
Factor model	7104.104	935	< .001

Additional fit measures

Fit indices

Index	Value
Comparative Fit Index (CFI)	0.889
Tucker-Lewis Index (TLI)	0.878
Bentler-Bonett Non-normed Fit Index (NNFI)	0.878
Bentler-Bonett Normed Fit Index (NFI)	0.875
Parsimony Normed Fit Index (PNFI)	0.790
Bollen's Relative Fit Index (RFI)	0.862
Bollen's Incremental Fit Index (IFI)	0.890
Relative Noncentrality Index (RNI)	0.889

Information criteria

	Value
Log-likelihood	−282351.360
Number of free parameters	146.000
Akaike (AIC)	564994.720
Bayesian (BIC)	565783.567
Sample-size adjusted Bayesian (SSABIC)	565319.749

Metric	Value
Root mean square error of approximation (RMSEA)	0.063
RMSEA 90% CI lower bound	0.062
RMSEA 90% CI upper bound	0.065
RMSEA p-value	0.000
Standardized root mean square residual (SRMR)	0.042
Hoelter's critical N (α = .05)	233.667
Hoelter's critical N (α = .01)	240.894
Goodness of fit index (GFI)	0.831
McDonald fit index (MFI)	0.153
Expected cross validation index (ECVI)	4.507

Section S5: Supplementary Hierarchical *g* and Horn no-*g* WJ III 46-test CFA

Two supplementary CFA models were completed. The results are presented here but are not interpreted in depth in this paper. As mentioned in the paper, the factor loadings of the first-order factors on the hierarchical *g* factor and the latent factor correlations in the Horn no-*g* models were helpful in evaluating the validity of the Gustafsson Gf=*g* model when compared to the Carroll 3S model.

Hierarchical *g* CFA model. The final McGrew-Carroll CFA model presented in Table 1 (in main manuscript) was used as the starting point for the specification of a hierarchical *g* model. The 10 first-order factors were specified as per the results in Table 1. The first-order *g* factor was removed. All 10 first-order factors were specified to load on a second-order (hierarchical) *g* factor. On the first run, the Sound Awareness test, which had been removed from the bifactor CFA, was added back into the model as an indicator of Ga. The resulting Ga factor had a near unity loading on *g* (.96), which was not consistent with the extant literature. It is possible that the Sound Awareness, because it is comprised of four different subtests, may be a factorially complex measure that functions as a mini proxy for *g*. Thus, Sound Awareness was again removed from the model resulting in a more reasonable Ga factor loading on *g* of .76. Modification indices were inspected and only two cross-loadings were added that made substantive sense (Story Recall on Gf; Retrieval Fluency on Gc).

The results of the hierarchical *g* model are presented in Table S4. The model fit statistics immediately follow in Table S5.

Table S4

WJ III 46-test Hierarchical g CFA Model

Test name	TM CHC	Gc	Grw	Gq	Gwm	Gl[1]	Gf	Gv	Ga	Gsa[2]	Gsc[2]
Verbal Comprehension	Gc	0.95									
General Information	Gc	0.91									
Academic Knowledge	Gc	0.88									
Reading Vocabulary	*Grw*	0.84									
Picture Vocabulary	Gc	0.83									
Oral Comprehension	Gc	0.68									
Passage Comprehension	*Grw*	0.67									
Story Recall	*Gl*	0.59					0.32				
Letter-Word Identification	Grw		0.83								
Word Attack	Grw		0.77								
Spelling	Grw		0.76								
Editing	Grw		0.76								
Writing Samples	Grw		0.68								
Spelling of Sounds	Grw		0.54						0.36		
Number Series[3]	Gf			0.84							
Number Matrices[3]	Gf			0.82							
Applied Problems	Gq			0.82							
Calculation	Gq			0.69							
Auditory Working Memory	Gwm				0.83						
Numbers Reversed	Gwm				0.72						
Memory for Words	Gwm				0.71						
Memory for Sentences	Gwm	0.25			0.53						
Visual-Auditory Learning	Gl					0.85					
Memory for Names	Gl					0.71					
Picture Recognition	*Gv*					0.50					
Visual Closure	*Gv*					0.30					
Concept Formation	Gf						0.89				
Understanding Directions	*Gwm*						0.78				0.27
Analysis-Synthesis	*Gf*			0.50			0.34				
Spatial Relations	Gv							0.68			
Block Rotation	Gv							0.65			
Planning	Gv							0.50			
Sound Blending	Ga								0.78		
Incomplete Words	Ga								0.61		
Sound Patterns-Voice	Ga								0.55		
Auditory Attention	Ga								0.47		0.22
Reading Fluency	*Grw*									0.64	0.44
Writing Fluency	*Grw*									0.45	0.38
Math Fluency	*Gq*			0.14						0.28	0.50
Pair Cancellation	Gs										0.88
Visual Matching	Gs										0.83
Cross Out	Gs										0.77
Decision Speed	Gs										0.75
Rapid Picture Naming	Gr									0.27	0.50
Retrieval Fluency	Gr	0.34									0.35
1st-order loadings on 2nd-order *g*		0.89	0.88	0.88	0.85	0.83	0.83	0.77	0.76	0.67	0.56

Note. TM CHC = CHC classifications from WJ III technical manual. Italics font indicates different CHC factor classification based on analysis. Factors ordered by 1st-order loadings on 2nd-order *g* factor. Tests ordered within 1st-order factors by magnitude of loading on primary CHC factor. [1] Original WJ III Glr factor classification changed to Gl or Gr and Gsm changed to Gwm as per McGrew et al. (2014) and Schneider and McGrew (2018). [2] Number Series and Number Matrices subtests were combinded as single Numerical Reasoning test in the WJ III.

Table S5

WJ III 46-test Hierarchical g CFA Model (Table S4) Fit Statistics

Model fit

Chi-square test

Model	X^2	df	p
Baseline model	54584.794	990	
Factor model	7811.816	923	< .001

Additional fit measures

Fit indices

Index	Value
Comparative Fit Index (CFI)	0.871
Tucker-Lewis Index (TLI)	0.862
Bentler-Bonett Non-normed Fit Index (NNFI)	0.862
Bentler-Bonett Normed Fit Index (NFI)	0.857
Parsimony Normed Fit Index (PNFI)	0.799
Bollen's Relative Fit Index (RFI)	0.846
Bollen's Incremental Fit Index (IFI)	0.872
Relative Noncentrality Index (RNI)	0.871

Information criteria

	Value
Log-likelihood	−277291.132
Number of free parameters	112.000
Akaike (AIC)	554806.264
Bayesian (BIC)	555411.407
Sample-size adjusted Bayesian (SSABIC)	555055.602

Other fit measures

Metric	Value
Root mean square error of approximation (RMSEA)	0.067
RMSEA 90% CI lower bound	0.066
RMSEA 90% CI upper bound	0.069
RMSEA p-value	0.000
Standardized root mean square residual (SRMR)	0.052
Hoelter's critical N (α = .05)	209.972
Hoelter's critical N (α = .01)	216.504
Goodness of fit index (GFI)	0.820
McDonald fit index (MFI)	0.123
Expected cross validation index (ECVI)	4.897

Horn no-*g* CFA model. The hierarchical *g* model presented in Table S4 was used as the starting point for the specification of a Horn no-*g* model. The 10 first-order factors were specified as per the results in Table S4. The second-order (hierarchical) *g* factor was removed and all 10 latent factors specified as obliquely correlated. The first run produced a negative latent factor correlation between the two speed factors (Gsc, Gsa). Thus, these two factors were collapsed into a single Gs factor. Only modifications indices (MI) related to the tests on the original Gsc and Gsa factors were considered. Based on MI's and substantive considerations, additional Grw cross-loadings were specified for Reading Fluency and Math Fluency. The results of the Horn no-*g* model are presented in Table C.3. The latent factor correlations are presented in Table S7. The model fit statistics immediately follow Table S8.

Table S6

WJ III 46-test Horn CFA no-g Model

Test name	TM CHC	Gc	Grw	Gq	Gwm	Gl[1]	Gf	Gv	Ga	Gs
Verbal Comprehension	Gc	0.95								
General Information	Gc	0.91								
Academic Knowledge	Gc	0.88								
Reading Vocabulary	*Grw*	0.84								
Picture Vocabulary	Gc	0.83								
Oral Comprehension	Gc	0.68								
Passage Comprehension	*Grw*	0.67								
Story Recall	*Gl*	0.59					0.32			
Letter-Word Identification	Grw		0.82							
Editing	Grw		0.77							
Spelling	Grw		0.76							
Word Attack	Grw		0.75							
Writing Samples	Grw		0.68							
Spelling of Sounds	Grw		0.51							0.39
Applied Problems	Gf			0.83						
Number Series[2]	Gf			0.83						
Number Matrices[2]	Gq			0.81						
Calculation	Gq			0.70						
Auditory Working Memory	Gwm				0.83					
Numbers Reversed	Gwm				0.73					
Memory for Words	Gwm				0.71					
Memory for Sentences	Gwm	0.29			0.50					
Visual-Auditory Learning	Gl					0.84				
Memory for Names	Gl					0.71				
Picture Recognition	*Gv*					0.50				
Visual Closure	*Gv*					0.31				
Concept Formation	Gf						0.89			
Understanding Directions	*Gwm*						0.78			0.26
Analysis-Synthesis	*Gf*			0.49			0.34			
Spatial Relations	Gv							0.66		
Block Rotation	Gv							0.66		
Planning	Gv							0.50		
Sound Blending	Ga								0.77	
Incomplete Words	Ga								0.61	
Sound Patterns-Voice	Ga								0.54	
Auditory Attention	Ga				0.22				0.46	
Pair Cancellation	Gs									0.86
Visual Matching	Gs									0.83
Cross Out	Gs									0.77
Decision Speed	Gs									0.74
Rapid Picture Naming	*Gr*									0.63
Math Fluency	*Gq*			0.27						0.57
Reading Fluency	*Grw*		0.43							0.49
Retrieval Fluency	*Gr*	0.33								0.38
Writing Fluency	*Grw*		0.41							0.35

Note. TM CHC = CHC classifications from WJ III technical manual. Italics font indicates different CHC factor classification based on analysis. Factors ordered like Table C.1 for comparability purposes. Tests ordered within factors by magnitude on primary CHC factor. Latent factor correlations are presented in Table C3. [1] Original WJ III Glr factor classification changed to Gl or Gr and Gsm changed to Gwm as per McGrew et al. (2014) and Schneider and McGrew (2018). [2] Number Series and Number Matrices were combined as a single Numerical Reasoning test in the WJ III.

Table S7

WJ III 46-test Horn no-g CFA Model (Table S6) Latent Factor Correlations

	Grw	Gc	Gl	Gs	Gf	Gwm	Gq	Gv	Ga
Grw	1.00								
Gc	0.83	1.00							
Gl	0.68	0.75	1.00						
Gs	0.55	0.43	0.48	1.00					
Gf	0.66	0.73	0.77	0.47	1.00				
Gwm	0.78	0.71	0.70	0.57	0.69	1.00			
Gq	0.79	0.79	0.72	0.52	0.77	0.72	1.00		
Gv	0.59	0.65	0.74	0.45	0.72	0.65	0.74	1.00	
Ga	0.70	0.69	0.65	0.45	0.63	0.78	0.56	0.61	1.00

Table S8

WJ III 46-test Horn no-g CFA Model (Table S6) Fit Statistics

Chi-square test

Model	X²	df	p
Baseline model	53504.285	946	
Factor model	7350.288	857	< .001

Additional fit measures

Fit indices

Index	Value
Comparative Fit Index (CFI)	0.876
Tucker-Lewis Index (TLI)	0.864
Bentler-Bonett Non-normed Fit Index (NNFI)	0.864
Bentler-Bonett Normed Fit Index (NFI)	0.863
Parsimony Normed Fit Index (PNFI)	0.781
Bollen's Relative Fit Index (RFI)	0.848
Bollen's Incremental Fit Index (IFI)	0.877
Relative Noncentrality Index (RNI)	0.876

Information criteria

	Value
Log-likelihood	−270794.519
Number of free parameters	133.000
Akaike (AIC)	541855.038
Bayesian (BIC)	542573.645
Sample-size adjusted Bayesian (SSABIC)	542151.126

Other fit measures

Metric	Value
Root mean square error of approximation (RMSEA)	0.068
RMSEA 90% CI lower bound	0.067
RMSEA 90% CI upper bound	0.069
RMSEA p-value	0.000
Standardized root mean square residual (SRMR)	0.046
Hoelter's critical N (α = .05)	207.784
Hoelter's critical N (α = .01)	214.487
Goodness of fit index (GFI)	0.828
McDonald fit index (MFI)	0.138
Expected cross validation index (ECVI)	4.641

Section S6: Carroll's Final Recorded Hand Printed Thoughts Regarding the General Intelligence (*g*) factor

Upon return from his May 2003 work week with Carroll, his daughter and Kevin McGrew exchanged several emails concerning the disposition of Carroll's massive collection of historically rich and well-catalogued correspondence with other giants in the field of intelligence. She would also send McGrew, from time to time, handwritten notes from Carroll that were intended to provide guidance on issues to address in a planned Carroll and McGrew coauthored revision of Carroll's 3S chapter originally published in Flanagan et al. (1997) and a planned coauthored paper of the 46-variable Carroll EFA analyses presented in this paper. A copy of one of these handwritten notes (dated 6-11-03) reflected Carroll's thinking regarding the concept of general intelligence (*g*). By this time Carroll's printing was hard to decipher. He would typically have a family member read his handwritten notes and query him regarding words or phrases that were difficult to decipher.They would insert the correct words or phrases. These clarifications are designated with boxes in a copy of the original note. Below is a transcription of Carroll's thoughts, followed by an image of the best available surviving copy of the 6-11-03 hand printed note.

This communication is presented "as is" for historical purposes so intelligence scholars might ponder, discuss, dissect, and debate. Carroll's reference to the Nyborg chapter is to his last formal publication (Carroll, 2003). Based on Carroll's many publications where he discussed the psychometric *g* factor, as well as this note, it is obvious Carroll believed that psychometric *g* was front-and-center in his thinking regarding the structure of human intelligence.

<u>*Points to emphasize in Kevin's elaboration on my chapter remarks.*</u>

The "proof" of G. It is important to recognize that in my paper published in the Nyborg book occurs the first modern, real scientific proof of G—to contrast by the many unacceptable "proofs" claimed by Spearman, Burt, Pearson, and others. It used the features of a complete proof advanced by LISREL technology. Jöreskog has discussed these features in his many writings. I suggest that you (Kevin) review his features and list them, contrasting them with features of incomplete proofs afforded by proposals of Burt, Pearson, and others.

Of particular interest are the proofs of the status of G, Gc and Gf, as provided in the Nyborg chapter. It was taken as accepted that these data were published in the Nyborg chapter--In the sense that G, Gc, and Gf could be independently established, plus several other factors (e.g. Gv, Ga). It was truly marvelous that enough data from these factors had accumulated to make their independence specifiable. Evidence now seems to be accumulating about the existence, distribution and meaning [of] the factors G, Gf, Gc and others.

G ("the general factor") appear[s] to pertain only to very "general" items of general knowledge—e.g. items of knowledge that are common to most people present only as specified by parameters of "item difficulty." G thus appears not to pertain to the many items of knowledge incorporated in Gf or Gc. These items of knowledge are in some way special—classified under Gf or Gc (or some combination of these). It appears that a human being becomes a "member of human society" only by acquiring aspects of special knowledge (either fluid or crystallized, or some

combination of them) (look at information on "item difficulties" -- in compilations of such information). (find literature review on this), (more special factors beyond Gf and Gc?)

Obviously my information is very incomplete.

6-22-03

POINTS TO EMPHASIZE IN KEVIN'S elaboration OF MY remarks

THE "PROOF" OF G. IT IS IMPORTANT TO RECOGNIZE THAT IN MY PAPER PUBLISHED IN THE NYBORG BOOK OCCURS THE first MODERN, REAL, SCIENTIFIC PROOF OF G. — IN CONTRAST TO THE MANY unacceptable "PROOFS" claimed BY SPEARMAN, BURT, PEARSON, AND OTHERS. IT USED THE FEATURES OF A complete PROOF advanced BY LISREL TECHNOLOGY. Jöreskog HAS DISCUSSED THESE FEATURES IN HIS MANY WRITINGS. I SUGGEST THAT YOU (KEVIN) REVIEW THESE features AND list THEM, CONTRASTING THEM WITH FEATURES OF INCOMPLETE PROOFS ADVANCED BY PROPOSALS OF BURT, PEARSON, AND OTHERS.

OF PARTICULAR INTEREST ARE THE PROOFS of THE STRUCTURES OF G, GC, AND GF, AS PROVIDED IN THE NYBORG CHAPTER. IT WAS taken as accepted THAT THESE DATA WERE PUBLISHED IN THE NYBORG CHAPTER — IN THE SENSE THAT G, GC, & GF COULD BE INDEPENDENTLY ESTABLISHED, PLUS SEVERAL OTHER FACTORS (e.g. Gv, Ga). IT WAS TRULY MARVELOUS THAT ENOUGH DATA ABOUT THESE FACTORS HAD ACCUMULATED TO MAKE THEIR INDEPENDENCE SPECIFIABLE

EVIDENCE NOW SEEMS TO BE ACCUMULATING ABOUT
THE EXISTENCE, DISTRIBUTION AND MEANING THE ~~[illegible]~~ FACTORS
G, GF, GC, and others.
G ("THE GENERAL FACTOR") APPEAR TO PERTAIN
ONLY TO VERY 'GENERAL' ~~ITEMS~~ OF GENERAL KNOWLEDGE, — E.G.
ITEMS OF KNOWLEDGE THAT ARE COMMON TO MOST PEOPLE,
PRESENT ONLY AS SPECIFIED BY PARAMETERS OF "ITEM
DIFFICULTY". G THUS APPEARS NOT TO PERTAIN TO
THE MANY ITEMS OF KNOWLEDGE INCORPORATED IN GF OR
GC. THESE ITEMS OF KNOWLEDGE ARE IN SOME WAY
SPECIAL- CLASSIFIED UNDER GF OR GC (BY SOME COMBINATION COMBINATION
OF THESE). IT APPEARS THAT A HUMAN BEING ~~PERSON~~ BECOMES A
"MEMBER OF HUMAN SOCIETY" ONLY BY ACQUIRING (HUMAN) ASPECTS OF SPECIAL
KNOWLEDGE (EITHER FLUID OR CRYSTALLIZED), IN SOME COMBINATION
OF THEM) (LOOK AT INFORMATION ON "ITEM
DIFFICULTIES" IN COMPILATIONS OF SUCH INFORMATION)
(FIND LITERATURE REVIEW ON THIS), (MORE
SPECIAL FACTORS BEYOND GF & GC?

OBVIOUSLY MY INFORMATION IS VERY
INCOMPLETE.

Section S7: CHC Higher-Stratum Design Psychometric Network Analysis Centrality Metrics and Weight Matrices for Reading and Math Models

Table S9

CHC Cognitive-Achievement PNA Centrality Metrics for Reading and Math Models

Reading model

Variable	Betweenness	Closeness	Strength
Cognitive			
GC3	0.00	0.81	0.66
GF3	1.00	1.00	1.00
GS3	0.60	0.74	0.68
GA3	0.00	0.69	0.67
GV2	0.00	0.68	0.44
GWM3	0.20	0.83	0.75
GL2	0.00	0.65	0.52
GR2	0.00	0.61	0.47
Achievement			
GRWR2	0.30	0.96	0.85

Math model

Variable	Betweenness	Closeness	Strength
Cognitive			
GC3	0.09	0.71	0.57
GF3	1.00	1.00	1.00
GS3	0.55	0.67	0.61
GA3	0.00	0.72	0.59
GV2	0.00	0.67	0.38
GWM3	0.18	0.86	0.65
GL2	0.00	0.61	0.46
GR2	0.00	0.55	0.42
Achievement			
GQ2	0.18	0.86	0.56

Table S10

CHC Cognitive-Achievement PNA Weights Matrices for Reading and Math Models

Weights matrix--reading model

Variable	GC3	GF3	GS3	GA3	GV2	GWM3	GL2	GR2	GRWR2
Cognitive									
GC3	0.00	0.22	0.00	0.11	0.00	0.04	0.08	0.01	0.35
GF3	0.22	0.00	0.13	0.05	0.26	0.16	0.21	0.00	0.22
GS3	0.00	0.13	0.00	0.00	0.04	0.13	0.02	0.39	0.14
GA3	0.11	0.05	0.00	0.00	0.08	0.26	0.09	0.09	0.14
GV2	0.00	0.26	0.04	0.08	0.00	0.04	0.13	0.00	0.01
GWM3	0.04	0.16	0.13	0.26	0.04	0.00	0.07	0.06	0.18
GL2	0.08	0.21	0.02	0.09	0.13	0.07	0.00	0.03	0.01
GR2	0.01	0.00	0.39	0.09	0.00	0.06	0.03	0.00	0.00
Achievement									
GRWR2	0.35	0.22	0.14	0.14	0.01	0.18	0.01	0.00	0.00

Weights matrix--math model

Variable	GC3	GF3	GS3	GA3	GV2	GWM3	GL2	GR2	GQ2
Cognitive									
GC3	0.00	0.20	0.00	0.17	0.01	0.12	0.08	0.03	0.20
GF3	0.20	0.00	0.11	0.08	0.23	0.18	0.17	0.00	0.42
GS3	0.00	0.11	0.00	0.03	0.04	0.16	0.02	0.39	0.11
GA3	0.17	0.08	0.03	0.00	0.08	0.28	0.10	0.08	0.00
GV2	0.01	0.23	0.04	0.08	0.00	0.04	0.12	0.00	0.01
GWM3	0.12	0.18	0.16	0.28	0.04	0.00	0.07	0.06	0.00
GL2	0.08	0.17	0.02	0.10	0.12	0.07	0.00	0.03	0.04
GR2	0.03	0.00	0.39	0.08	0.00	0.06	0.03	0.00	0.00
Achievement									
GQ2	0.20	0.42	0.11	0.00	0.01	0.00	0.04	0.00	0.00

References

Carroll, John B. 1993. *Human Cognitive Abilities: A Survey of Factor-Analytic Studies*. Cambridge: Cambridge University Press. https://doi.org/10.1017/cbo9780511571312.

Carroll, John B. 2003. The higher-stratum structure of cognitive abilities: Current evidence supports *g* and about ten broad factors. In *The Scientific Study of General Intelligence: Tribute to Arthur R. Jensen*. Edited by Helmuth Nyborg. Oxford: Pergamon, pp. 5–22.

Schneider, W. Joel, and Kevin S. McGrew. 2018. The Cattell-Horn-Carroll theory of cognitive abilities. In *Contemporary Intellectual Assessment: Theories, Tests and Issues*, 4th ed. Edited by Dawn P. Flanagan and Erin M. McDonough. New York: Guilford Press, pp. 73–130.
